# Alcohol consumption in relation to carotid subclinical atherosclerosis and its progression: results from a European longitudinal multicentre study

**DOI:** 10.1007/s00394-020-02220-5

**Published:** 2020-03-24

**Authors:** Federica Laguzzi, Damiano Baldassarre, Fabrizio Veglia, Rona J. Strawbridge, Steve E. Humphries, Rainer Rauramaa, Andries J. Smit, Philippe Giral, Angela Silveira, Elena Tremoli, Anders Hamsten, Ulf de Faire, Paolo Frumento, Karin Leander, IMPROVE Study group, C. R. Sirtori, S. Castelnuovo, M. Amato, B. Frigerio, A. Ravani, D. Sansaro, C. Tedesco, D. Coggi, A. Bonomi, M. J. Eriksson, J. Cooper, J. Acharya, K. Huttunen, E. Rauramaa, H. Pekkarinen, I. M. Penttila, J. Törrönen, A. I. van Gessel, A. M. van Roon, G. C. Teune, W. D. Kuipers, M. Bruin, A. Nicolai, P. Haarsma-Jorritsma, D. J. Mulder, H. J. G. Bilo, G. H. Smeets, J. L. Beaudeux, J. F. Kahn, V. Carreau, A. Kontush, J. Karppi, T. Nurmi, K. Nyyssönen, R. Salonen, T. P. Tuomainen, J. Tuomainen, J. Kauhanen, G. Vaudo, A. Alaeddin, D. Siepi, G. Lupattelli, E. Mannarino

**Affiliations:** 1 Unit of Cardiovascular and Nutritional Epidemiology, Institute of Environmental Medicine, Karolinska Institutet, Nobels väg 13, Box 210, 17177 Stockholm, Sweden; 2 Department of Medical Biotechnology and Translational Medicine, Università degli Studi di Milano, Milan, Italy; 3 Centro Cardiologico Monzino, IRCCS, Milan, Italy; 4 Institute of Mental Health and Wellbeing, Mental Health and Wellbeing, University of Glasgow, Glasgow, UK; 5 Cardiovascular Medicine Unit, Department of Medicine, Karolinska Institutet, Stockholm, Sweden; 6 Centre for Cardiovascular Genetics, Institute Cardiovascular Science, University College London, London, UK; 7 Foundation for Research in Health Exercise and Nutrition, Kuopio Research Institute of Exercise Medicine, Kuopio, Finland; 8 Department of Clinical Physiology and Nuclear Medicine, Kuopio University Hospital, Kuopio, Finland; 9 Department of Medicine, University Medical Centre Groningen, University of Groningen, Groningen, The Netherlands; 10 Assistance Publique-Hôpitaux de Paris, Service Endocrinologie-Métabolisme, Groupe Hospitalier Pitié-Salpétrière, Unités de Prévention Cardiovasculaire, Paris, France; 11 Department of Cardiology, Karolinska University Hospital, Stockholm, Sweden; 12 Unit of Biostatistics, Institute of Environmental Medicine, Karolinska Institutet, Nobels väg 13, 17177 Stockholm, Sweden

**Keywords:** Alcohol drinking, Atherosclerosis, Carotid intima-media thickness, Progression, Epidemiology

## Abstract

**Background/Aim:**

The association between alcohol consumption and subclinical atherosclerosis is still unclear. Using data from a European multicentre study, we assess subclinical atherosclerosis and its 30-month progression by carotid intima-media thickness (C-IMT) measurements, and correlate this information with self-reported data on alcohol consumption.

**Methods:**

Between 2002–2004, 1772 men and 1931 women aged 54–79 years with at least three risk factors for cardiovascular disease (CVD) were recruited in Italy, France, Netherlands, Sweden, and Finland. Self-reported alcohol consumption, assessed at baseline, was categorized as follows: none (0 g/d), very-low (0 − 5 g/d), low (> 5 to  ≤ 10 g/d), moderate (> 10 to ≤ 20 g/d for women,  > 10 to ≤ 30 g/d for men) and high (> 20 g/d for women, > 30 g/d for men). C-IMT was measured in millimeters at baseline and after 30 months. Measurements consisted of the mean and maximum values of the common carotids (CC), internal carotid artery (ICA), and bifurcations (Bif) and whole carotid tree. We used quantile regression to describe the associations between C-IMT measures and alcohol consumption categories, adjusting for sex, age, physical activity, education, smoking, diet, and latitude.

**Results:**

Adjusted differences between median C-IMT values in different levels of alcohol consumption (vs. very-low) showed that moderate alcohol consumption was associated with lower C-IMT_max_[− 0.17(95%CI − 0.32; − 0.02)], and Bif-IMT_mean_[− 0.07(95%CI − 0.13; − 0.01)] at baseline and decreasing C-IMT_mean_[− 0.006 (95%CI − 0.011; − 0.000)], Bif-IMT_mean_[− 0.016(95%CI − 0.027; − 0.005)], ICA-IMT_mean_[− 0.009(95% − 0.016; − 0.002)] and ICA-IMT_max_[− 0.016(95%: − 0.032; − 0.000)] after 30 months. There was no evidence of departure from linearity in the association between alcohol consumption and C-IMT.

**Conclusion:**

In this European population at high risk of CVD, findings show an inverse relation between moderate alcohol consumption and carotid subclinical atherosclerosis and its 30-month progression, independently of several potential confounders.

**Electronic supplementary material:**

The online version of this article (10.1007/s00394-020-02220-5) contains supplementary material, which is available to authorized users.

## Introduction

The relation between alcohol consumption and atherosclerosis is still far from established. Atherosclerosis, the main cause of cardiovascular disease (CVD), is a complex chronic low–grade inflammatory disease involving accumulation of lipids and inflammatory markers in the arteries [1, 2]. Measurements of intima-media thickness in the carotid artery (C-IMT), assessed through simple, non-invasive diagnostic techniques, are considered valid indicators of subclinical atherosclerosis as well as of risk of incident CVD [3]. Low-moderate alcohol consumption, corresponding to no more than three standard glasses per day in men and two in women, has previously been shown to exert anti-inflammatory, anti-oxidant, fibrinolytic, and lipid-lowering effects, and to decrease the risk of CVD [4–7]. In contrast, higher alcohol consumption has been associated with increased inflammation, oxidation, and increased risk of CVD [4, 8].

Findings from epidemiological studies investigating the association between alcohol consumption and C-IMT have shown inconsistent results: some found a protective effect of moderate alcohol consumptions [9–20], others suggested that alcohol is always a risk factor [21–26], and yet others showed no association [27–35]. Some of the studies have described the relationship between alcohol consumption and atherosclerosis as linear, with either increased [22, 25] or decreased C-IMT [13, 16] associated with a rise in alcohol consumption, whereas others report a J-shaped association, with a decrease of C-IMT with moderate alcohol consumption and an increase of C-IMT with high alcohol consumption [9, 14, 15, 17]. Few studies, mainly performed in men [23, 24, 27, 28], often with heavy or binge drinking habits [23, 24, 27], have investigated the relationship between alcohol consumption and progression of atherosclerosis, and results were discrepant [12, 23, 24, 27, 28, 36].

We aimed to investigate the relationship between alcohol consumption and subclinical atherosclerosis and its 30-month progression in a European multi-centre study including middle-aged men and women at high risk of CVD.

## Methods

### Study population

The Carotid Intima Media Thickness (IMT) and IMT-PROgression as Predictors of Vascular Events in a High-Risk European Population study (IMPROVE) is a European multi-centre study including middle-aged men (*n* = 1772) and women (*n* = 1931) with at least three CVD risk factors. From 2002 to 2004, participants were recruited from seven different centres located in: Italy (two centres: Milan and Perugia), France (Paris), the Netherlands (Groningen), Sweden (Stockholm) and Finland (two centres in Kuopio). The study complies with the Declaration of Helsinki and was approved by the Institutional Review Board of each centre. All patients gave written informed consent. A detailed description of the IMPROVE study is reported elsewhere [37, 38].

The present study was conducted in accordance with the STROBE guidelines [39].

### Alcohol consumption assessment

At baseline, participants were asked to recall their daily consumption of alcoholic beverages in ml (considering that one glass of wine ≈ 200 ml, a pint of beer ≈ 570 ml and a can of beer ≈ 330 ml) and spirits (one glass of spirit ≈ 25 ml). From these data, total alcohol consumption per day (g/day) was calculated, considering the different content of alcohol in wine, beer and spirits. We created five categories of alcohol: none (0 g/day), very low [(0, 5) g/day], low [(5, 10) g/day], moderate [(10, 20) g/day for women and (10, 30) g/day for men] and high (> 20 g/day for women and > 30 g/d for men). These categories were created to capture approximately none, half, one, two–three, and above three standard glasses per day, respectively. One standard glass is normally defined as containing 8-12 g of alcohol and correspond to alcohol content in one bottle of beer (330 ml), one glass of wine (120 ml), or one glass of spirits (40 ml) [40]. Nineteen participants (11 men and 8 women) with missing information on alcohol consumption were excluded from the analyses.

### Carotid IMT measurements

C-IMT, expressed in millimetres (mm), were measured at baseline and after 30 months, by B-mode ultrasonography. For this study, we considered the average of the mean (IMT_mean_) and the maximum (IMT_max_) of the C-IMT measured in the whole carotid arteries and in specific segments i.e. common (CC-IMT_mean_, CC-IMT_max_), bifurcation (Bif-IMT_mean_, Bif-IMT_max_) and internal (ICA-IMT_mean_, ICA-IMT_max_). The 30-month progression was expressed as mean difference between the 30-month measurement and baseline C-IMT divided for the follow-up time (mm/year). Details of the method and its validation are reported elsewhere [37, 38]. For the progression analysis, 422 participants who dropped out during the follow-up period were excluded.

### Possible confounders

Smoking status was dichotomized in never- and ever-smoker (current or former smoker). Physical activity was categorized into three groups: low (brisk walk for 10 min less than once a week), medium (brisk walk for 10 min at least two–three times/week) and high (brisk walk for 10 min more than three times/week). Education level was categorized into three groups: less than 9 years of school (compulsory school), 9–12 years of school (secondary) and > 12 years of school (university or college). A score reflecting dietary habits, from 0 to 5 corresponding to level of adherence to a healthy diet, was created as the sum of various dietary items. In details, one point was assigned for each of the following dietary habits which were regarded as “healthy”: olive oil as main source of type of fat consumed, fish intake more than two times per week, meat intake less than 2 times per week, three or more fruits per day and milk less than 4 dl/day. Based on the recruitment centres, latitude was categorized into six different groups capturing North–South geographical gradient; for descriptive purpose a binary variable (North/South) was created, categorized according to a previous publication [37] Sex and age were also considered as potential confounders.

### Statistical methods

As descriptive statistics, we report the median and the interquartile range (IQR) for continuous variables, and the sample proportions (%) for categorical variables.

Quantile regression (QR) models at the 50th (p50, median) and 75th percentiles (p75, 3rd quartile) were employed to evaluate the association between alcohol categories and C-IMT measurements at baseline and after 30 months. The rationale for choosing this statistical approach is that it allows the analyst to regress any percentile of the outcome distribution including median and the high percentiles (75th) of the C-IMT [41]. In this population with right skewed C–IMT, the mean values would not provide information on the right tail of the distribution that can also capture abnormal C-IMT indicative of high risk of CVD [42]. Results are delivered as regression coefficients with 95% confidence intervals (CI). The regression coefficients are interpreted as the 50th and 75th percentile differences in the response variable (a C-IMT measurement) between a specific category of alcohol consumption and the reference category, that corresponds to very low alcohol consumption. Models were adjusted for sex and age (Model 1) plus physical activity, smoking, diet, latitude and education level (Model 2).

To understand the shape of the association between alcohol consumption and the selected percentiles of C-IMT, we also estimated a variation of Model 2 in which we employed restricted cubic splines with four knots at 4, 10, 20 and 30 g/day to model the effect of alcohol consumption. In this analysis, alcohol consumption was treated as a continuous variable, allowing for a nonlinear effect. These analyses were performed only for those associations that were observed to be significant in the main model. To assess departure from linearity, we tested the nullity of the coefficients associated with the second, third and fourth spline basis.

To verify the robustness of the results, we further adjusted Model 2 for potential mediators of the effect of alcohol consumption on C-IMT. The factors included in the model were: body mass index, high density lipoproteins (HDL), lipid lowering treatments (defined as use of fibrates, statins, omega-3 and resins and used as a proxy for hypercholesterolemia), hypertension (defined as anamnestic or use of antihypertensive treatment or SBP ≥ 140 mmHg or DBP ≥ 90 mmHg) and diabetes (defined as self-reported, or use of anti-diabetic medicine or blood glucose > 7 mmol/l).

Based on previous knowledge of sex-specific biological mechanisms in atherosclerosis [43] and that patterns of alcohol consumption and alcohol metabolism vary by sex [44], we performed additional analyses in which men and women were investigated separately. Previous literature on sex-specific associations between alcohol consumption and subclinical atherosclerosis is scarce, in particular in regard to progression.

Sensitivity analyses were performed excluding participants with a CVD event occurring between the time of enrolment and the visit after 30 months.

**Fig. 1:**
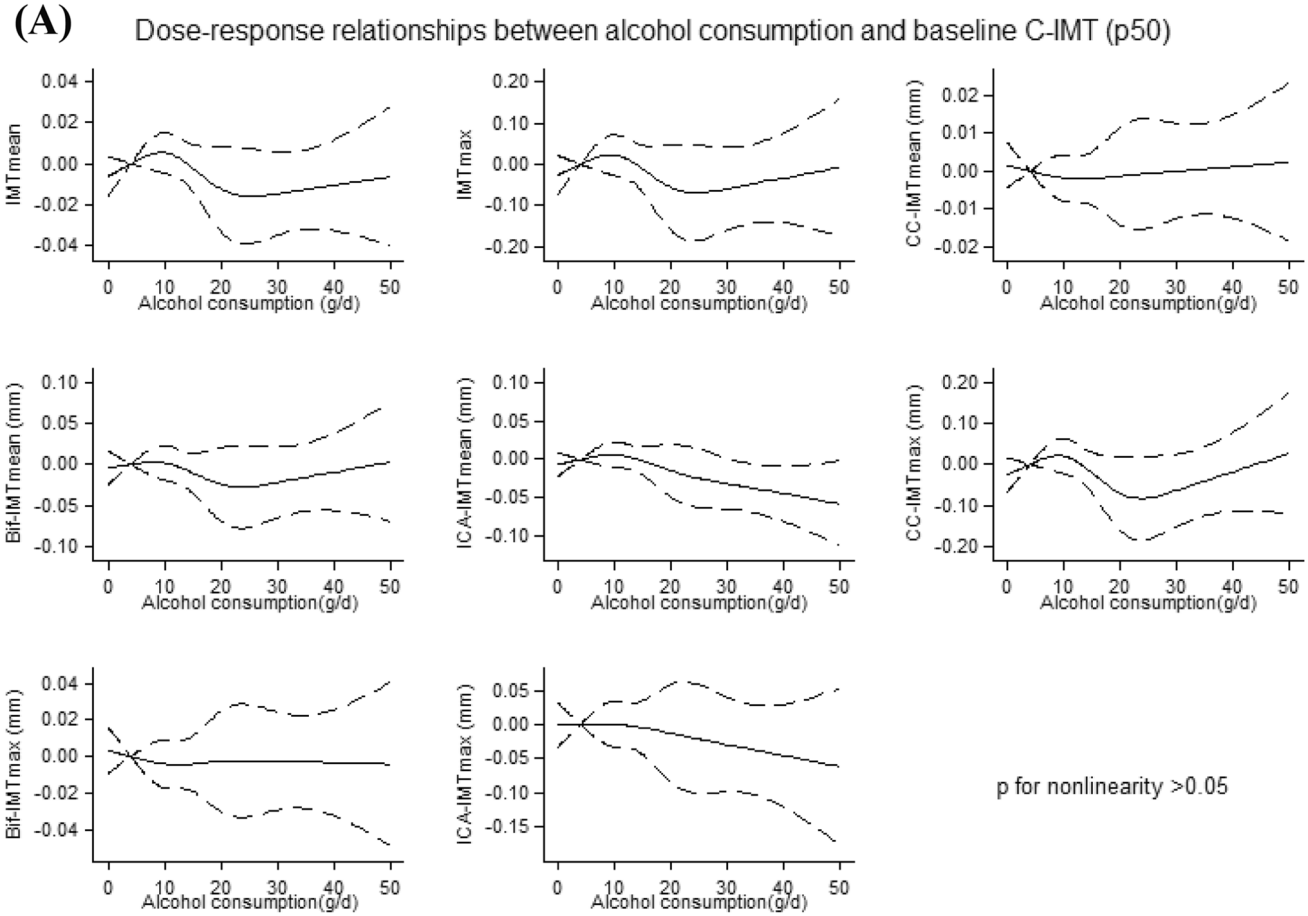

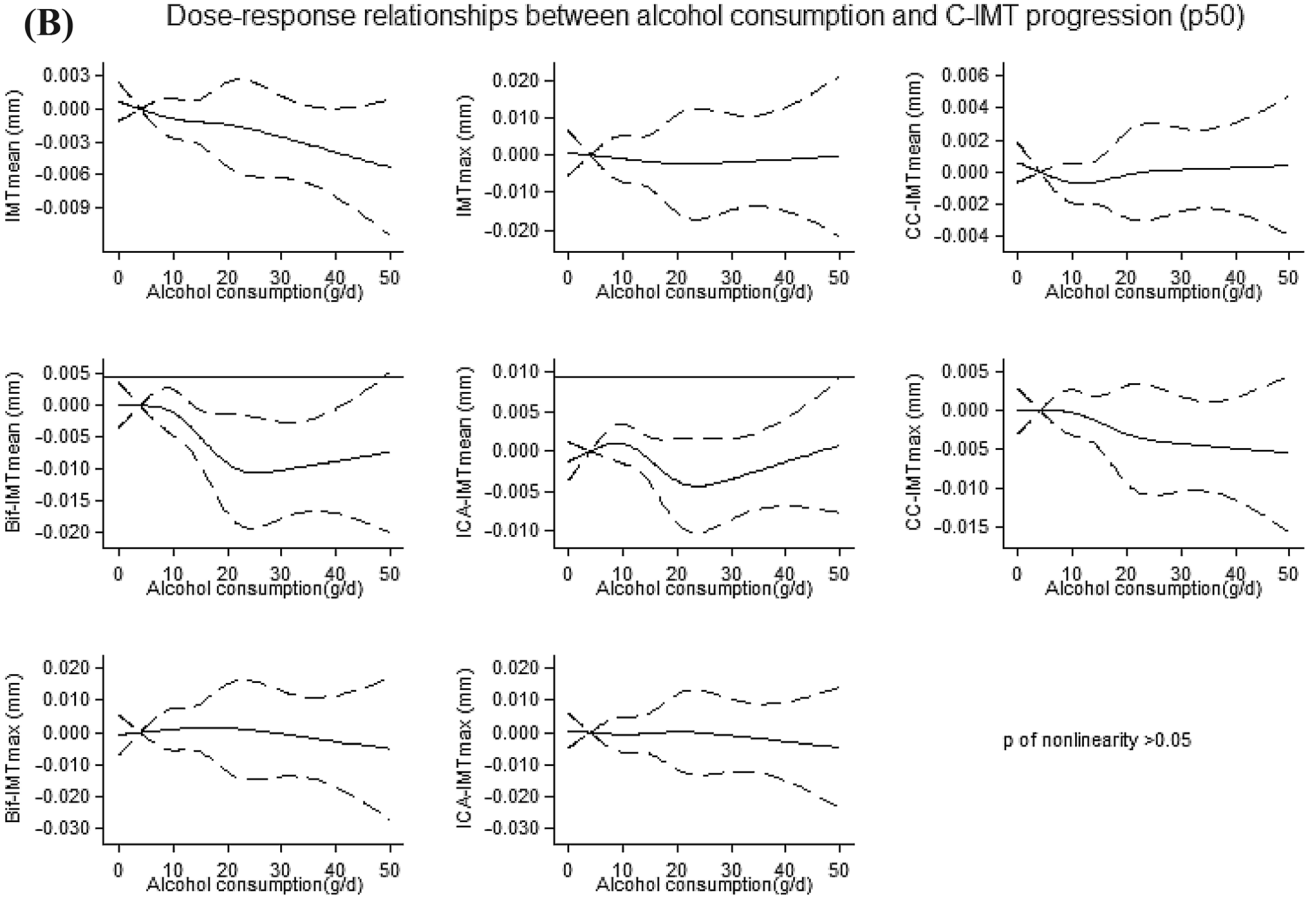
**a**, **b** Dose–response relationships between alcohol consumption and each of the considered measurements of C-IMT (p50) at baseline (**a**) and progression (**b**). Solid lines: Restricted cubic splines adjusted for sex, age, physical activity, smoking, diet, and latitude, with knots located at fixed points of g/d of alcohol consumption (4, 10, 20, 30). Dashed lines: 95% CI. 4 g/day was used as a reference point. P for nonlinearity was obtained testing the nullity of the coefficients associated with the second, third and fourth spline basis. For a better readability of the graphs, we excluded participants with alcohol consumption > 50 g/d

Missing data were handled by exclusion from each analysis. The total amount of missing data on covariates was less than 4% for baseline and progression analysis, respectively. A flowchart of the study participants is presented in Figure 1, Supplementary Materials.

Statistical analyses were performed using STATA software (STATA version 12.1, Corp, College Station, TX, USA).

## Results

Table 1 shows the distribution of descriptive characteristics of the IMPROVE participants included in this study (*n* = 3684) and in men and women, separately. The majority of the participants reported no alcohol consumption (*n* = 1678), driven mainly by the large proportion of non-consumers in women (69%). Most of the physically active and non-smoking participants, respectively, had very low alcohol consumption whereas the highly educated more often had a moderate or high alcohol consumption.

**Table 1:** Baseline characteristics by different levels of alcohol consumption of IMPROVE study participants

Characteristic	Abstainers (0 g/d)	Very Low (> 0–5 g/d)	Low (> 5–10 g/d)	Moderate (> 10–30 g/d)^a^	High (> 30 g/d)^b^
*n*					
All	1678	225	375	738	668
Men	515	119	179	468	480
Women	1163	106	196	270	188
Total alcohol (g/d)					
All	0 (0;0)	4 (1.9;4)	8 (8;8)	16 (16;16.8)	36 (32;48)
Men	0 (0;0)	3.6 (2;4)	8 (8;8)	16 (16;21.6)	40 (32;56)
Women	0 (0;0)	4 (1.8;4)	8 (8;8)	16 (16;16)	32 (32;33)
Age (y)					
All	64.4 (59.6;67.3)	65.3 (60.5;67.4)	65.6 (60;67.2)	65.2 (59.5;67.2)	63.4 (59.1;67)
Men	64.6 (59.5;67.1)	65.3 (59.9;67.3)	64.9 (59.3;67.3)	65.7 (59.3;67.2)	63.2 (59.1;66.9)
Women	64.2 (59.7;67.5)	65.2 (61.4;67.8)	65.9 (60.1;67.1)	65 (59.8;67.2)	63.6 (59.4;67.4)
Physical activity (%) ^m3^					
All					
Low	22.3	8.4	16.3	18.0	21.6
Medium	43.7	42.2	42.8	42.9	49.4
High	34.0	49.3	40.9	39.0	29.0
Men					
Low	16.0	7.6	14.0	13.7	20.2
Medium	42.6	41.2	41.6	40.2	49.4
High	41.4	51.3	44.4	46.1	30.4
Women					
Low	25.0	9.0	18.0	25.6	25.0
Medium	44.2	43.0	44.0	47.8	49.5
High	30.7	47.0	38.0	26.7	25.5
Ever smoker (%)					
All	13.3	10.2	14.7	15.7	19.2
Men	14.9	10.1	19.0	16.7	18.9
Women	12.6	10.4	10.7	14.1	19.7
Education (%) ^m34^					
All					
≤ 9 years	51.6	44.6	46.4	39.6	38.5
9 − 12 years	25.3	25.2	23.2	26.3	27.0
> 12 years	23.0	30.2	30.5	34.1	34.4
Men					
≤ 9 years	44.9	50.0	44.6	37.1	37.2
9 − 12 years	25.2	23.7	21.5	24.3	27.3
> 12 years	30.0	26.3	33.9	38.6	35.5
Women					
≤ 9 years	54.5	38.5	47.9	43.8	41.9
9 − 12 years	25.5	26.9	24.7	29.7	26.3
> 12 years	20.0	34.6	27.3	26.4	31.7
Dietscore^c m14^					
All	2 (1;3)	1(0;2)	2 (1;3)	2 (1;3)	2 (1;3)
Men	1(1;2)	1(1;2)	1(1;2)	1(1;2)	2 (1;3)
Women	2 (1;3)	1(0;2)	2 (1;3)	2 (1;3)	2 (2;3)
Geographical gradient (%)^d^					
All					
North	57.0	93.0	62.0	59.0	40.5
South	43.0	7.0	38.0	41.0	59.0
Men					
North	66.0	97.0	76.0	70.0	44.0
South	34.0	3.0	24.0	30.0	56.0
Women					
North	53.0	88.7	48.9	40.0	32.0
South	47.0	11.0	49.0	60.0	67.5
Lipid-lowering drugs (%)^e m63^					
All	49.0	43.0	50.0	45.0	54.5
Men	46.0	43.0	43.0	44.0	55.0
Women	51.0	44.0	56.0	47.5	54.3

Results are presented for all the participants (*n* = 3684), in men (*n* = 1761) and in women (*n* = 1923), respectively. Median and interquartile range (in brackets) for continuous variables where not specified; proportions for binary and categorical variables (%)

*m* missing values

^a^For women cut-off > 10** − ** < 20 g/day

^b^For women cut-off > 20 g/day

^c^Dietscore continuous variable created as described in the Method section

^d^North includes Finland (2 centers in Kuopio), Sweden (Stockholm), The Netherlands (Groningen); South: France (Paris), Italy (1 center in Milan, 1 center in Perugia)

^e^Hypolipidemic treatment including statins, fibrate, resins

Hypertension was common among very low consumers of alcohol, and hypertriglyceridemia was common among high consumers. Uric acid was higher among moderate and high consumers, and adiponectin was higher among low consumers. Slightly higher concentrations of total cholesterol and Low Density Lipoprotein (LDL), but not HDL, were also found among moderate and high consumers (vs very low) (Table 1, Supplementary Materials).

Results from analyses of association between alcohol consumption and median C-IMT at baseline are presented in Table 2. When compared to a very low consumption, moderate, high and no alcohol consumption were associated with lower IMT_max_. Further, moderate alcohol consumption was associated with lower Bif-IMT_mean_. These results were independent of confounders included in Model 2. No clear associations were found for alcohol consumption and IMT_mean_, ICA-IMT_mean._ and ICA-IMT_max_ measured at baseline.

**Table 2:** Median differences (95% CI) of IMT measured at baseline in relation to alcohol consumption categories

IMT Baseline		Abstainers (0 g/d)	Very low (> 0 − 5 g/d)	Low (> 5–10 g/d)	Moderate (> 10–30 g/d)^a^	High (> 30 g/d)^b^
		*n* = 1,678	*n* = 225	*n* = 375	*n* = 738	*n* = 668
	Models	*β*_1_ (95%CI)	Reference	*β*_1_ (95%CI)	*β*_1_ (95%CI)	*β*_1_ (95%CI)
IMT_mean_^m2^						
p50	Model 1	− 0.06 (− 0.09; − 0.03)	–	− 0.05 (− 0.09; − 0.01)	− 0.07 (− 0.1; − 0.04)	− 0.09 (− 0.12 ; − 0.06)
	Model 2	− 0.02 (− 0.05; 0.01)	–	0.00 (− 0.04; 0.03)	− 0.02 (− 0.05; 0.01)	− 0.02 (− 0.05; 0.01)
IMT_max_^m2^						
p50	Model 1	− 0.32(− 0.48; − 0.16)	–	− 0.25(− 0.43; − 0.06)	− 0.33(− 0.50; − 0.16)	− 0.39(− 0.56; − 0.22)
	Model 2	− 0.18 (− 0.32; − 0.04)	–	− 0.11 (− 0.27; 0.06)	− 0.17 (− 0.32; − 0.02)	− 0.16 (− 0.32; − 0.01)
CC–IMT_mean_^m4^						
p50	Model 1	− 0.02 (− 0.04; 0.00)	–	− 0.02 (− 0.04; 0.00)	− 0.03 (− 0.05; − 0.01)	− 0.03 (− 0.05; − 0.01)
	Model 2	0.00 (− 0.02; 0.02)	–	0.00 (− 0.02; 0.02)	− 0.01 (− 0.03; 0.01)	0.00 (− 0.02; 0.02)
Bif–IMT_mean_^m2^						
p50	Model 1	− 0.12 (− 0.18; − 0.07)	–	− 0.08 (− 0.15; − 0.01)	− 0.16 (− 0.22; − 0.10)	− 0.15 (− 0.21; − 0.09)
	Model 2	− 0.04 (− 0.10; 0.02)	–	0.00 (− 0.07; 0.06)	− 0.07 (− 0.13; − 0.01)	− 0.05 (− 0.11; 0.02)
ICA IMT_mean_^m34^						
p50	Model 1	− 0.05 (− 0.09; − 0.01)	–	− 0.05 (− 0.10; 0.00)	− 0.06 (− 0.11; − 0.02)	− 0.09 (− 0.14; − 0.05)
	Model 2	− 0.03 (− 0.07; 0.02)	–	− 0.03 (− 0.08; 0.02)	− 0.03 (− 0.07; 0.02)	− 0.05 (− 0.09; 0.00)
CC–IMT_max_^m4^						
p50	Model 1	− 0.04 (− 0.08; 0.01)	–	− 0.02 (− 0.07; 0.02)	− 0.04 (− 0.08; 0.00)	− 0.05 (− 0.10; − 0.01)
	Model 2	0.00 (− 0.04; 0.04)	–	0.01 (− 0.04; 0.05)	− 0.01 (− 0.05; 0.03)	0.00 (− 0.04; 0.04)
Bif–IMT_max_^m21^						
p50	Model 1	− 0.20 (− 0.33; − 0.08)	–	− 0.12 (− 0.27; 0.03)	− 0.25 (− 0.39; − 0.12)	− 0.29 (− 0.43; − 0.16)
	Model 2	− 0.04 (− 0.16; 0.08)	–	− 0.01 (− 0.15; 0.14)	− 0.06 (− 0.20; 0.07)	− 0.10 (− 0.23; 0.04)
ICA IMT_max_^m34^						
p50	Model 1	− 0.12 (− 0.22; − 0.01)	–	− 0.11 (− 0.23; 0.01)	− 0.17 (− 0.28; − 0.06)	− 0.17 (− 0.28; − 0.06)
	Model 2	0.00 (− 0.10; 0.10)	–	0.00 (− 0.12; 0.12)	− 0.02 (− 0.13; 0.09)	− 0.01 (− 0.13; 0.10)

Results for all participants of the IMPROVE study (*n* = 3684). Number of observations for each analysis: IMT_mean and_ IMT_max_: Model 1, *n* = 3682; Model 2, *n* = 3635; CC-IMT_mean_ and CC-IMT_max_: Model 1, *n* = 3680; Model 2, *n* = 3633; Bif-IMT_mean_ and Bif-IMT_max_: Model 1, *n* = 3663; Model 2, *n* = 3616; ICA-IMT_mean_ and ICA-IMT_max_: Model 1, *n* = 3650; Model 2, *n* = 3603

*Model 1* Adjustments for sex and age; *Model 2* Model 1 plus physical activity, education, smoking, latitude (categorical) and diet (continuous); *m* missing values

^a^For women cut-off > 10 to  < 20 g/day

^b^For women cut-off > 20 g/day

The associations between alcohol consumption and median C-IMT progression are shown in Table 3. When compared to a very low consumption, any consumption of alcohol (low, moderate and high) was associated with lower IMT_mean_ progression. Moreover, moderate and high alcohol consumption were associated with lower Bif-IMT_mean_, ICA-IMT_mean._ and ICA-IMT_max_ progression. These results remained significant after the adjustments in Model 2. For the progression, no associations were found for IMT_max_ and CC-IMT.

**Table 3:** Median differences (95% CI) of C-IMT progression in relation to alcohol consumption categories

	IMT progression		Abstainers (0 g/d)	Very low (> 0 − 5 g/d)	Low (> 5–10 g/d)	Moderate (> 10 − 30 g/d)^a^	High (> 30 g/d)^b^
		Models	n = 1,471	*n* = 209	*n* = 332	*n* = 658	*n* = 592
			*β*_1_ (95%CI)	Ref	*β*_1_ (95%CI)	*β*_1_ (95%CI)	*β*_1_ (95%CI)
	IMT_mean_^m10^						
	p50	Model 1	− 0.004 (− 0.009; 0.001)	–	− 0.009 (− 0.016; − 0.003)	− 0.008 (− 0.013; − 0.002)	− 0.008 (− 0.014; − 0.002)
		Model 2	− 0.005 (− 0.001; − 0.000)	–	− 0.007 (− 0.013; − 0.001)	− 0.006 (− 0.011; − 0.000)	− 0.008 (− 0.014; − 0.002)
	IMT_max_^m2^						
	p50	Model 1	0.004 (− 0.011; 0.019)	–	− 0.010 (− 0.028; 0.008)	0.000 (− 0.016; 0.016)	− 0.001 (− 0.018; 0.015)
		Model 2	0.007 (− 0.011; 0.025)	–	− 0.001 (− 0.022; 0.020)	0.011 (− 0.008; 0.030)	0.002 (− 0.018; 0.022)
	CC–IMT_mean_^m2^						
	p50	Model 1	0.000 (− 0.004; 0.004)	–	− 0.003 (− 0.007; 0.002)	− 0.003 (− 0.008; 0.001)	− 0.002 (− 0.006; 0.002)
		Model 2	− 0.001 (− 0.005; 0.002)	–	− 0.002 (− 0.006; 0.002)	− 0.002 (− 0.006; 0.002)	− 0.002 (− 0.006; 0.002)
	Bif–IMT_mean_^m13^						
	p50	Model 1	− 0.012 (− 0.022; − 0.002)	–	− 0.016 (− 0.028; − 0.004)	− 0.020 (− 0.031; − 0.009)	− 0.021 (− 0.032; − 0.01)
		Model 2	− 0.010 (− 0.020; 0.001)	–	− 0.011 (− 0.023; 0.001)	− 0.016 (− 0.027; − 0.005)	− 0.016 (− 0.027; − 0.004)
	ICA IMT_mean_^m20^						
	p50	Model 1	− 0.010 (− 0.016; − 0.004)	–	− 0.007 (− 0.014; 0.000)	− 0.011 (− 0.017; − 0.005)	− 0.011 (− 0.017; − 0.004)
		Model 2	− 0.008 (− 0.015; − 0.001)	–	− 0.005 (− 0.012; 0.003)	− 0.009 (− 0.016; − 0.002)	− 0.008 (− 0.015; − 0.001)
	CC–IMT_max_^m2^						
	p50	Model 1	0.004 (− 0.004; 0.012)	–	0.003 (− 0.007; 0.013)	− 0.002 (− 0.011; 0.007)	− 0.004 (− 0.013; 0.005)
		Model 2	0.004 (− 0.005; 0.012)	–	0.003 (− 0.007; 0.013)	0.000 (− 0.009; 0.009)	− 0.003 (− 0.012; 0.006)
	Bif–IMT_max_^m13^						
	p50	Model 1	0.001 (− 0.015; 0.017)	–	0.000 (− 0.020; 0.019)	− 0.001 (− 0.019; 0.016)	0.000 (− 0.018; 0.017)
		Model 2	0.004 (− 0.014; 0.022)	–	0.005 (− 0.016; 0.027)	0.004 (− 0.016; 0.024)	0.002 (− 0.018; 0.023)
	ICA IMT_max_^m20^						
	p50	Model 1	− 0.020 (− 0.034; − 0.006)	–	− 0.022 (− 0.039; − 0.005)	− 0.020 (− 0.036; − 0.005)	− 0.028 (− 0.044; − 0.012)
		Model 2	− 0.016 (− 0.031; − 0.002)	–	− 0.017 (− 0.034; 0.000)	− 0.016 (− 0.032; − 0.000)	− 0.022 (− 0.038; − 0.006)

Results for all participants of the IMPROVE study for whom follow-up data on C-IMT were available (*n* = 3262). Number of observation for each analysis: IMT_mean,_ Model 1,*n* = 3252; Model 2, *n* = 3211; IMT _max_, CC-IMT _mean_ and CC-IMT_max_: Model 1, *n* = 3260; Model 2, *n* = 3219; Bif-IMT_mean_ and Bif-IMT_max_: Model 1, *n* = 3249; Model 2, *n* = 3208; ICA-IMT_mean_ and ICA-IMT_max_: Model 1, *n* = 3242; Model 2, *n* = 3201

*Model 1* Adjustments for sex and age; *Model 2*: Model 2 plus physical activity, education, smoking, latitude (categorical) and diet (continuous); *m* missing values

^a^For women cut-off > 10 to  < 20 g/day

^b^For women cut-off > 20 g/day

No departure from linearity (*p* > 0.05) was found for the associations between alcohol consumption and median C-IMT at baseline (Fig. 1a) and progression (Fig. 1b).

Analysis of the association between alcohol consumption and the 75th percentile of C-IMT showed that moderate and no alcohol consumption were associated with lower CC-IMT_mean_ at baseline (Supplementary Material, Table 2) whereas no clear associations were found with C-IMT progression (Supplementary Material, Table 3). An indication of linearity was also shown for the dose–response relationships between alcohol consumption and the 75th percentile of C-IMT (Supplementary Material, Figure 1 A–B).

Results from multivariate analysis with additional adjustment for possible intermediate factors were still significant (data not shown), although the associations between moderate alcohol consumption and Bif-MT_mean_ [− 0.06 (− 0.13; 0.00)] and IMT_mean_ [− 0.005 (− 0.011; 0.000)] progression were slightly attenuated.

Analyses stratified by sex showed associations between alcohol consumption and C-IMT in the same direction as the main analysis (Supplementary Material, Tables 4, 5). Significant associations were found for moderate alcohol consumption and C-IMT_max_ and CC-IMT_mean,_ in men, at baseline, and C-IMT_mean_, ICA-IMT_mean_ and ICA-IMT_max_ in women for the progression. There was a clear relation between moderate alcohol consumption and Bif–IMT_mean_ progression both in men and women. However, results were limited by fairly low statistical power.

Regarding the exclusion of participants with CVD events occurring during the period between baseline and the measurements after 30th months (*n* = 215), results were consistent with the main analysis (data not shown).

## Discussion

In this European multi-centre study including participants at high risk of CVD but free of clinical manifestation of CVD at baseline, alcohol consumption was inversely associated, arguably in an approximately linear fashion, with subclinical carotid atherosclerosis and its 30-month progression. These results were independent of sex, age, physical activity, smoking, diet, education and latitude. In particular, compared to very low alcohol consumption, we found that moderate and high alcohol consumption were associated with a lower composite (C-IMT_mean_) and segment specific (bifurcation and internal carotid) C-IMT progression. At baseline, moderate alcohol consumption was associated with a lower composite (C-IMT_max_) and segment specific C-IMT (bifurcations). Lower C-IMT at baseline (C-IMT_max_) and progression (internal carotids) were also found for the abstainers.

Our findings of moderate alcohol consumption in relation to decreased C-IMT measured at baseline confirm the results of some earlier studies [9, 10, 13–17, 19, 21, 36] but not all [11, 25, 30, 32–34]. Among the few studies [12, 36] that have investigated the association between alcohol consumption and progression of atherosclerosis including both men and women, our study is one of the largest. Our findings of lower C-IMT progression in relation to moderate and high alcohol consumption, as compared to very low consumption, agree to some extent with those reported from an Italian study (*n* = 780) [12] but disagree with those of an American study (*n* = 788) [36]. The Italian study observed protective associations also for light-moderate alcohol consumption (50 g/day), compared to abstainers, in their case in relation to atherosclerotic plaque. Compared to our study, participants were healthier and the follow-up was longer (5 years) [12]. The American study was performed in individuals affected by HIV which may hamper comparability between studies due to presence of different confounding factors in the study base [36].

In contrast to previous studies that have found a linear increase [22, 25] or J-shaped curve for the association between alcohol consumption and C-IMT [9, 14, 15, 17], our findings support a linear decrease of C-IMT (both at baseline and after 30-month follow-up) in relation to increasing alcohol consumption. A linear decrease of IMT was previously reported in two other large epidemiological studies (*n* > 4000) including Korean men and women [13, 16]. The earlier investigations with opposite findings to our study were performed in Americans [15], Chinese [17], Finnish [25], Germans [14, 22] and Italians [9]. Apart from the study population origins being different, the intake of alcohol in our study was generally lower (median 4 g/d IQR: 0 − 16). In our study, only 4% of all the participants consumed more than 50 g/d (corresponding to more than 3 drinks per day), possibly explaining the discrepant findings. In addition, compared with the compared studies, our population was at higher risk of CVD; in subjects with metabolic disturbances and chronic low-grade inflammation, alcohol consumption may attenuate the effect of the risk factors for atherosclerosis [22]. Moreover, a large proportion of our study participants at high risk of CVD were under pharmacological poly-therapy (including drugs with pleiotropic effect such as statins) that may also have altered the effects of alcohol on C-IMT, regardless of the amount of alcohol consumed [45]. Nonetheless, when we controlled for lipid lowering treatment including statins, the associations were only slightly attenuated.

The biological mechanisms behind a potentially causal protective effect exerted by moderate alcohol consumption on subclinical atherosclerosis and CVD are not completely understood. Epidemiological and experimental studies have suggested that low–moderate (up to three standard drinks) doses of alcohol consumption may have a beneficial effect on the cascade of factors (e.g. lipoprotein, coagulation, adiponectin, inflammatory chemokines, vascular endothelial growth factors) that lead to the formation of atherosclerotic plaques [4, 5, 46, 47]. On the other hand, high alcohol consumption may drive the formation of higher amount of the toxic metabolite acetaldehyde. In turn, this may lead to the formation of biological markers involved in the development of the atherosclerotic process [4].

Our results of differential associations referred to different carotid segments, observed at baseline and progression and in men and women separately, are relatively complex to interpret. Carotid subclinical atherosclerosis measured in different segments has been suggested to have different clinical significance; CC-IMT may reflect hyperplasia or hypertrophy of smooth cells strongly related to age, whereas Bif-IMT and ICA-IMT may indicate a pathological response to low shear stress leading to the development of abnormal carotid atherosclerosis [48]. Also, CVD risk factors and atherosclerotic progression are more strongly associated with Bif-IMT and ICA-IMT than with CC-IMT [48]. We found a consistent protective association between alcohol and Bif-IMT (both at baseline and at progression), and a non-consistent association with CC-IMT and ICA-IMT. Both findings appear reasonable in the light of previous observations.

### Strengths and limitations

A strength of this study is that it is based on a unique cohort with a large sample size, including both men and women, and with availability of data from several C-IMT segments, allowing to capture different physiological and clinical profiles. Importantly, C-IMT measurements were validated and followed a common protocol for all centres. We cannot exclude, however, that some of the results could be false positives. However, the proportion of significant findings (36% at baseline, and 23% at progression) was much larger than the 5% false positive that could be expected by chance under the null hypothesis.

Our results showed robustness against additional adjustment for CVD risk factors. Obviously, we cannot exclude that other possible unmeasured and/or unknown factors that we have not controlled for may explain the observed associations.

Another strength of our study is that we used as reference category the very low consumers; low alcohol consumption has lately been considered a more appropriate group of comparison than abstainers [49, 50]. It is possible that the group of abstainers includes a number of former drinkers who quit due to the presence of comorbidity or metabolic disorder. Such situation would contribute to explain the finding of a lower C-IMT at baseline and at progression for abstainer in comparison to low consumers.

Our study has also some limitations. Alcohol consumption was self-reported and we had no possibility to validate the reported intake of alcohol. Misclassifications may have led to non-differential misclassification of exposure, diluting the estimated effects. Moreover, we do not have repeated measures of alcohol consumption, so we were not able to detect possible changes over the 30-month follow-up.

Although the study is representative of the European population with classical CVD risk factors, the inclusion of different European countries with different drinking patterns may have introduced heterogeneity in the results. Nordic countries are for example known to have a more binge drinking pattern than the Southern European countries. We adjusted for latitude but we were not able to stratify by countries due to lack of statistical power. However, when we stratified by north and south geographical location of centres, results were similar (data not shown).

We cannot rule out the presence of bias due to non-participation at follow-up. However, the mean alcohol consumption was similar in the missing group (mean 12.0 g/day sd. 18 g/day) as compared to the participant group (mean 12.3 g/day sd. 18 g/day) making selection bias less likely to affect the internal validity of our study.

Finally, the follow-up for progression of atherosclerosis was fairly short (30 months). However, in an experimental study in mice, a clear decrease of atherosclerotic plaque was already observed after 2 weeks, for daily moderate drinking [51].

## Conclusion

In this study population at high risk of CVD, moderate alcohol consumption was inversely associated with measurements of C-IMT and its progression. This finding supports the hypothesis of a vascular protective effect exerted by moderate alcohol consumption. However, for clinical implications, it is important to consider that moderate alcohol consumption may increase risk of other diseases such as cancer.

## Electronic supplementary material

Below is the link to the electronic supplementary material.

Supplementary file1 (DOCX 393 kb)
