# Supplementary material for: Alcohol consumption in relation to carotid subclinical atherosclerosis and its progression: results from a European longitudinal multicentre study
Source: Eur J Nutr. 2020 Mar 24;60(1):123–34. doi: 10.1007/s00394-020-02220-5 (PMC7867553; doi:10.1007/s00394-020-02220-5)
Supplement: Supplementary file 1 — Supplementary file1 (DOCX 393 kb) [file 394_2020_2220_MOESM1_ESM.docx]

Figure 1: Flowchart of the participants included in the study.

Table 1: Distribution of classical and novel cardio-metabolic variables by levels of alcohol consumption in the participants of the IMPROVE study (n=3,684). Results are presented for all the participants (n=3,684), in men (n=1,761) and in women (n=1,923), respectively.

| **Characteristic** | **Abstainers**  **(0g/d)** | **Very Low**  **(0−5 g/d)** | **Low**  **(>5−10g/d)** | **Moderate**  **(>10−30g/d) ^a^** | **High**  **(>30g/d) ^b^** |
| --- | --- | --- | --- | --- | --- |
| Hypertension (%) |  |  |  |  |  |
| All | 80 | 87 | 78 | 80 | 76 |
| Men | 78 | 81.5 | 80 | 83 | 76 |
| Women | 81 | 93 | 77 | 74 | 75.5 |
| Hypercholesterolemia (%) ^m4^ |  |  |  |  |  |
| All | 68.2 | 66.7 | 70.1 | 69.2 | 74.4 |
| Men | 56.8 | 59.6 | 57.5 | 64.0 | 71.5 |
| Women | 73.3 | 74.5 | 81.6 | 78.1 | 81.9 |
| Hypertriglyceridemia (%) ^m2^ |  |  |  |  |  |
| All | 25.6 | 11.6 | 21.6 | 22.4 | 36.2 |
| Men | 31.9 | 13.4 | 27.9 | 25.8 | 38.9 |
| Women | 22.8 | 9.4 | 15.8 | 16.3 | 29.3 |
| S-Total cholesterol (mmol/L) ^m15^ |  |  |  |  |  |
| All | 5.4 (4.7;6.2) | 5.1 (4.5;5.9) | 5.4 (4.7;6.3) | 5.5 (4.8;6.3) | 5.6 (4.8;6.3) |
| Men | 5.1(4.5;5.8) | 4.9 (4.4;5.6) | 5.1 (4.4;5.8) | 5.3 (4.7;5.9) | 5.4 (4.6;6.1) |
| Women | 5.6 (4.8;6.3) | 5.3 (4.7;6.1) | 5.8 (5;6.6) | 5.8 (5.1;6.5) | 6.1 (5.3;6.7) |
| S-HDL (mmol/L)^m16^ |  |  |  |  |  |
| All | 1.2 (1.0;1.5) | 1.2 (1.0;1.5) | 1.3 (1;1.5) | 1.2 (1.0;1.5) | 1.2 (1.0;1.5) |
| Men | 1.0 (0.8;1.2) | 1.1 (1.0;1.3) | 1.1 (0.9;1.3) | 1.1 (0.9;1.3) | 1.1 (0.9;1.4) |
| Women | 1.3 (1.1;1.5) | 1.4 (1.1;1.6) | 1.4 (1.2;1.7) | 1.4 (1.1;1.7) | 1.4 (1.2;1.6) |
| S-LDL (mmol/L)^m79^ |  |  |  |  |  |
| All | 3.5 (2.8;4.2) | 3.2 (2.6;4.0) | 3.4 (2.8;4.2) | 3.5 (2.9;4.2) | 3.5 (2.8;4.3) |
| Men | 3.3 (2.3;3.9) | 3.2 (2.6;3.7) | 3.2 (2.6;3.9) | 3.5 (2.8;4.1) | 3.5 (2.8;4.2) |
| Women | 3.6 (2.9;4.3) | 3.2 (2.6;4.1) | 3.7 (2.9;4.5) | 3.8 (3.0;4.4) | 3.8 (3.1;4.6) |
| S-Triglycerides (mmol/L)^m15^ |  |  |  |  |  |
| All | 1.3 (0.9;1.9) | 1.2 (0.9;1.7) | 1.3 (0.9;1.7) | 1.2 (0.9;1.8) | 1.5 (1.0;2.0) |
| Men | 1.4 (1.0;2.0) | 1.2 (0.9;1.7) | 1.3 (0.9;1.9) | 1.3 (0.9;2.0) | 1.5 (1.0;2.1) |
| Women | 1.3 (0.9;1.9) | 1.2 (0.9;1.6) | 1.2 (0.9;1.6) | 1.2 (0.8;1.6) | 1.4 (0.9;1.9) |
| Diabetes (%) ^m63^ |  |  |  |  |  |
| All | 29.0 | 24.0 | 25.0 | 23.0 | 28.0 |
| Men | 35.0 | 30.0 | 35.0 | 29.0 | 31.0 |
| Women | 26.0 | 18.0 | 16.0 | 13.5 | 18.0 |
| S/P-Glucose (mmol/L) ^m12^ |  |  |  |  |  |
| All | 5.5 (4.9;6.2) | 5.7 (5.3;6.5) | 5.6 (5;6.4) | 5.6 (5.0;6.4) | 5.6 (5.0;6.3) |
| Men | 5.6 (5.1;6.5) | 5.9 (5.4;6.8) | 5.8 (5.3;6.7) | 5.8 (5.17;6.7) | 5.7 (5.1;6.5) |
| Women | 5.3 (4.8;6.1) | 5.5 (5.1;6.3) | 5.3 (4.8;6.0) | 5.2 (4.8;5.9) | 5.3 (4.8;5.8) |
| S-Uric acid (mmol/L) ^m17^ |  |  |  |  |  |
| All | 297.5 (254.5;345.2) | 306.6 (265.8;350.8) | 308.2 (260.8;356.2) | 317.0 (271.6;368.9) | 334.1 (284.0;392.0) |
| Men | 331.9 (285.1;381.5) | 330.1 (293.8;361.1) | 337.9 (295.7;383.7) | 335.7 (298.4;384.1) | 344.6 (298.4;404.0) |
| Women | 284.3 (245.7;328.7) | 278.7 (236.2;324.2) | 282.7 (245.2;323.9) | 276.5 (235.4;324.2) | 296.1 (248.1;347.4) |
| S-CRP (mmol/L) ^m11^ |  |  |  |  |  |
| All | 2.0 (0.8;3.9) | 1.4 (0.5;3.4) | 1.6 (0.7;3.5) | 1.7 (0.8;3.2) | 1.9 (0.8;3.5) |
| Men | 1.8 (0.7;3.5) | 1.2 (0.5;2.6) | 1.4 (0.5;3.0) | 1.6 (0.7;3.0) | 1.8 (0.7;3.4) |
| Women | 2.1 (0.9;4.0) | 1.7 (0.7;4.0) | 2.1 (0.9;4.0) | 2.1 (0.9;3.5) | 2.0 (0.9;3.8) |
| BMI (Kg/m2) ^m3^ |  |  |  |  |  |
| All | 27.0 (24.2;30.1) | 27.1 (24.3;29.7) | 26.3 (24.0;28.7) | 26.6 (24.5;29.1) | 27,0 (24.7;29.3) |
| Men | 27.0 (24.9;29.1) | 27.1 (24.4;29.4) | 27.2 (25.1;29.1) | 26.8 (24.9;29.2) | 27.3(25.3;29.4) |
| Women | 27.0 (23.8;30.4) | 26.6 (24.1;30.3) | 25.6 (23.0;28.3) | 25.8 (23.4;28.6) | 25.8 (23.2;28.7) |
| S-Creatinine (umol/l) ^m16^ |  |  |  |  |  |
| All | 75.2 (66.4;87.2) | 82.05 (71.9;93.7) | 79.5 (67.6;90.6) | 81.3 (71.9;93.5) | 84.3 (72.5;94.1) |
| Men | 89.1 (79.5;100.0) | 90 (79.7;99.8) | 88.8 (80.4;99.5) | 88.1 (79.2;98.3) | 88.3 (80.2;97.7) |
| Women | 70.7 (62.9;79.3) | 73.2 (65.2;82.1) | 70.1 (62.1;79.15) | 70.5 (63.4;79.5) | 69.3 (61.8;79.6) |
| P-Adiponectin (µg/mL) ^m13^ |  |  |  |  |  |
| All | 11.7 (6.54;17.8) | 7.8 (4.0;13.7) | 11.0 (6.4;18.1) | 10.3 (6.2;17.5) | 10.0 (6.2;16.0) |
| Men | 7.9 (4.5;11.3) | 6.0 (3.2;10.6) | 7.2 (4.6;11.0) | 8.2 (5.0;12.3) | 8.7 (5.27;13.3) |
| Women | 13.1(8.1;20.7) | 11.1 (5.4;20.2) | 16.3 (10.5;23.6) | 17.3 (10.9;24.4) | 15.9 (9.7;21.8) |
| P-IgG antiApoB ^m14^ |  |  |  |  |  |
| All | 0.8 (0.7;1.0) | 0.8 (0.7;1.0) | 0.9 (0.7;1.1) | 0.8 (0.7;1.0) | 0.82 (0.6;1.0) |
| Men | 0.8 (0.6;1.1) | 0.8 (0.6;1.0) | 0.9 (0.7;1.1) | 0.8 (0.7;1.0) | 0.81 (0.6;1.0) |
| Women | 0.8 (0.7;1.0) | 0.8 (0.7;1.1) | 0.9 (0.7;1.0) | 0.9 (0.7;1.0) | 0.8 (0.7;1.0) |
| P-IgM antiApoB ^m14^ |  |  |  |  |  |
| All | 0.97 (0.84;1.04) | 0.98 (0.86;1.05) | 0.98 (0.85;1.05) | 0.98 (0.8;1.0) | 1.0 (0.8;1.0) |
| Men | 0.96 (0.78;1.04) | 0.94 (0.83;1.05) | 0.97 (0.83;1.05) | 1.0 (0.8;1.0) | 1.0 (0.8;1.0) |
| Women | 0.97 (0.86;1.04) | 1 (0.88;1.04) | 0.99 (0.87;1.04) | 1.0 (0.8;1.0) | 1.0 (0.9;1.0) |
| S-Anti−PC (U/mL) ^m13^ |  |  |  |  |  |
| All | 65.0 (41.0;102) | 63.0 (36.0;104.0) | 62.0 (43.0;99.0) | 64 (39;103) | 62.0 (38.0;98.0) |
| Men | 59.0 (35.0;102.5) | 570 (32.0;102.0) | 58.0 (37.0;89.0) | 59 (36;98) | 59 (37.0;93.0) |
| Women | 67.0 (42.0;102.0) | 69.5 (42.0;110.0) | 72.0 (48.0;106.0) | 72 .0.0(43;111) | 68.0 (44.0;108.0) |
| P-Leptin (ng/ mL) ^m13^ |  |  |  |  |  |
| All | 19.1(10.1;33.45) | 13.2 (6.7;26.1) | 13.7 (8.1;23.9) | 12.8 (7.2;22.3) | 11.4 (7.0;19.1) |
| Men | 8.9 (5.6;13.8) | 7.6 (5.2;11.9) | 8.6 (5.4;13.5) | 8.9 (6.2;13.5) | 9.3 (6.2;13.9) |
| Women | 26.3 (15.8;40.5) | 24.5 (15.7;38.6) | 22.5 (13.5;32.8) | 24.2 (17.3;36.0) | 23.6 (15.6;35.7) |
| P-IL-5 (pg/mL) ^m13^ |  |  |  |  |  |
| All | 0.4 (0.3;0.7) | 0.5 (0.3;0.8) | 0.5 (0.3;0.7) | 0.4 (0.3;0.7) | 0.42 (0.2;0.7) |
| Men | 0.5 (0.3;0.8) | 0.5 (0.3;0.9) | 0.5 (0.3;0.9) | 0.5 (0.3;0.7) | 0.4 (0.3;0.7) |
| Women | 0.4 (0.2;0.6) | 0.4 (0.3;0.7) | 0.4 (0.3;0.7) | 0.3 (0.3;0.6) | 0.3 (0.2;0.6) |
| P-CD93 (ng/mL) ^m240^ |  |  |  |  |  |
| All | 157.1 (135.1;184.8) | 155.7 (134.9;179.9) | 159.0 (135.1;183.3) | 154.8 (134.4;179.6) | 154.1 (130.2;185.0) |
| Men | 158.6 (136.1;185.6) | 155.1 (135.2;180.8) | 160.0(131.7;183.3) | 153.9 (134.3;180.9) | 153.6 (128.8;183.5) |
| Women | 156.1 (134.8;184.6) | 155.8 (133.6;179.0) | 158 (138.8;186.7) | 155.9 (134.8;179.04) | 155.4 (130.5;192.3) |

Median and interquartile range (in brackets) for continuous variables; proportions for binary and categorical variables (%)

S, serum; P, plasma; m, missing

^a^ for women cut-off >10**−**<20g/day;

^b^ for women cut-off>20g/day.

Table 2: 75^th^ percentile differences (95% CI) of IMT measured at baseline in relation to alcohol consumption categories. Results for all participants of the IMPROVE study (n=3,684).

| **IMT BASELINE** |  |  | **Abstainers**  **(0g/d)** | **Very Low**  **(>0−5 g/d)** | **Low**  **(>5−10g/d)** | **Moderate**  **(>10−30g/d) ^a^** | **High**  **(>30g/d) ^b^** |
| --- | --- | --- | --- | --- | --- | --- | --- |
|  |  |  | n=1,678 | n=225 | n=375 | n=738 | n=668 |
|  |  | **Models** | **β_1_ (95%CI)** | **Reference** | **β_1_ (95%CI)** | **β_1_ (95%CI)** | **β_1_ (95%CI)** |
| **IMT_mean_^m2^** |  |  |  |  |  |  |  |
|  | p75 | Model 1 | −0.06 (−0.1;−0.02) | − | −0.05 (−0.1;0.00) | −0.07 (−0.11;−0.02) | −0.06 (−0.11;−0.02) |
|  |  | Model 2 | −0.02 (−0.06;0.03) | − | 0.01 (−0.04;0.06) | −0.01 (−0.05;0.04) | −0.01 (−0.06;0.04) |
| **IMT_max_^m2^** |  |  |  |  |  |  |  |
|  | p75 | Model 1 | −0.36 (−0.53;−0.18) | − | −0.20 (−0.41;0.00) | −0.42 (−0.61;−0.23) | −0.30 (−0.49;−0.11) |
|  |  | Model 2 | −0.02 (−0.06;0.03) | − | 0.01 (−0.04;0.06) | −0.01 (−0.05;0.04) | −0.01 (−0.06;0.04) |
| **CC−IMT_mean_^m4^** |  |  |  |  |  |  |  |
|  | p75 | Model 1 | −0.04 (−0.07;−0.01) | − | −0.03 (−0.06;0.01) | −0.05 (−0.08;−0.02) | −0.04 (−0.07;−0.01) |
|  |  | Model 2 | −0.03 (−0.05;−0.00) | − | −0.02 (−0.05;0.01) | −0.04 (−0.07;−0.01) | −0.03 (−0.06;0.00) |
| **Bif−IMT_mean_^m21^** |  |  |  |  |  |  |  |
|  | p75 | Model 1 | −0.08 (−0.17;0.00) | − | −0.09 (−0.19;0.02) | −0.16 (−0.25;−0.06) | −0.17 (−0.27;−0.08) |
|  |  | Model 2 | −0.01 (−0.10;0.08) | − | 0.01 (−0.1;0.12) | −0.03 (−0.13;0.07) | −0.06 (−0.16;0.04) |
| **ICA IMT_mean_^m34^** |  |  |  |  |  |  |  |
|  | p75 | Model 1 | −0.09 (−0.17;−0.02) | − | −0.07 (−0.16;0.03) | −0.07 (−0.16;0.01) | −0.10 (−0.19;−0.02) |
|  |  | Model 2 | −0.04 (−0.12;0.05) | − | 0.01 (−0.10;0.11) | −0.03 (−0.12;0.07) | −0.01 (−0.11;0.08) |
| **CC−IMT_max_^m4^** |  |  |  |  |  |  |  |
|  | p75 | Model 1 | −0.06 (−0.14;0.02) | − | 0.02 (−0.07;0.12) | −0.08 (−0.16;0.01) | −0.04 (−0.12;0.05) |
|  |  | Model 2 | 0.00 (−0.08;0.09) | − | 0.05 (−0.05;0.15) | −0.01 (−0.10;0.08) | 0.02 (−0.07;0.11) |
| **Bif−IMT_max_^m21^** |  |  |  |  |  |  |  |
|  | p75 | Model 1 | −0.31 (−0.48;−0.14) | − | −0.20 (−0.40;0.01) | −0.35 (−0.53;−0.17) | −0.36 (−0.55;−0.18) |
|  |  | Model 2 | −0.13 (−0.30;0.05) | − | 0.01 (−0.19;0.21) | −0.14 (−0.32;0.05) | −0.16 (−0.36;0.03) |
| **ICA IMT_max_^m34^** |  |  |  |  |  |  |  |
|  | p75 | Model 1 | −0.14 (−0.33;0.05) | − | −0.04 (−0.27;0.18) | −0.13 (−0.33;0.07) | −0.15 (−0.35;0.06) |
|  |  | Model 2 | −0.01 (−0.21;0.20) | − | 0.02 (−0.22;0.27) | 0.00 (−0.23;0.22) | 0.03 (−0.20;0.26) |

Model 1: Adjustments for sex and age; Model 2: Model 1 plus physical activity, education, smoking, latitude (categorical) and diet (continuous);

m, missing values

^a^ for women cut-off >10**−**<20g/day;

^b^ for women cut-off>20g/day.

Number of observations for each analysis: IMT_mean_ and IMT_max_: Model 1, n=3,682; Model 2, n=3,635; CC-IMT_mean_ and CC-IMT_max_: Model 1, n=3,680; Model 2, n=3,633; Bif-IMT_mean_ and Bif-IMT_max_: Model 1, n=3,663; Model 2, n=3,616; ICA-IMT_mean_ and ICA-IMT_max_: Model 1, n=3,650; Model 2, n=3,603

Table 3: 75^th^ percentile differences (95% CI) of C-IMT progression in relation to alcohol consumption categories. Results for all participants of the IMPROVE study for whom follow-up data on C-IMT are available (n=3,262).

| **IMT progression** |  |  | **Abstainers**  **(0g/d)** | **Very Low**  **(>0−5 g/d)** | **Low**  **(>5−10g/d)** | **Moderate**  **(>10−30g/d) ^a^** | **High**  **(>30g/d) ^b^** |
| --- | --- | --- | --- | --- | --- | --- | --- |
|  |  |  | n=1,471 | n=209 | n=332 | n=658 | n=592 |
|  |  |  | **β_1_ (95%CI)** | **REF** | **β_1_ (95%CI)** | **β_1_ (95%CI)** | **β_1_ (95%CI)** |
| **IMT_mean_^m10^** |  |  |  |  |  |  |  |
|  | p75 | Model 1 | −0.005 (−0.011;0.001) | − | −0.006 (−0.014;0.001) | −0.010 (−0.016;−0.003) | −0.008 (−0.015;−0.002) |
|  |  | Model 2 | −0.002 (−0.009;0.004) | − | −0.002 (−0.009;0.006) | −0.005 (−0.012;0.002) | −0.004 (−0.011;0.004) |
| **IMT_max_^m2^** |  |  |  |  |  |  |  |
|  | p75 | Model 1 | 0.002 (−0.027;0.031) | − | −0.025 (−0.06;0.009) | −0.023 (−0.054;0.008) | −0.024 (−0.056;0.007) |
|  |  | Model 2 | 0.019 (−0.013;0.052) | − | −0.008 (−0.046;0.03) | 0.006 (−0.028;0.041) | 0.005 (−0.031;0.041) |
| **CC−IMT_mean_^m2^** |  |  |  |  |  |  |  |
|  | p75 | Model 1 | 0.00 (−0.004;0.005) | − | −0.002 (−0.007;0.004) | −0.004 (−0.009;0.001) | −0.003 (−0.008;0.002) |
|  |  | Model 2 | −0.000 (−0.005;0.004) | − | −0.004 (−0.009;0.002) | −0.003 (−0.008;0.002) | −0.003 (−0.008;0.003) |
| **Bif−IMT_mean_^m13^** |  |  |  |  |  |  |  |
|  | p75 | Model 1 | −0.007 (−0.021;0.007) | − | −0.010 (−0.027;0.006) | −0.019 (−0.034;−0.004) | −0.015 (−0.031;0.000) |
|  |  | Model 2 | −0.002 (−0.015;0.012) | − | −0.002 (−0.018;0.014) | −0.008 (−0.022;0.007) | −0.008 (−0.024;0.007) |
| **ICA IMT_mean_^m20^** |  |  |  |  |  |  |  |
|  | p75 | Model 1 | −0.010 (−0.021;0.001) | − | −0.009 (−0.022;0.004) | −0.014 (−0.025;−0.002) | −0.009 (−0.021;0.003) |
|  |  | Model 2 | −0.009 (−0.022;0.004) | − | −0.007 (−0.022;0.008) | −0.010 (−0.024;0.004) | −0.008 (−0.023;0.006) |
| **CC−IMT_max_^m2^** |  |  |  |  |  |  |  |
|  | p75 | Model 1 | −0.002 (−0.016;0.013) | − | −0.006 (−0.023;0.011) | −0.012 (−0.027;0.004) | −0.014 (−0.030;0.001) |
|  |  | Model 2 | 0.003 (−0.011;0.018) | − | 0.000 (−0.017;0.017) | −0.007 (−0.022;0.009) | −0.009 (−0.025;0.007) |
| **Bif−IMT_max_^m13^** |  |  |  |  |  |  |  |
|  | p75 | Model 1 | 0.001 (−0.031;0.033) | − | −0.021 (−0.059;0.017) | −0.020 (−0.054;0.014) | −0.017 (−0.052;0.018) |
|  |  | Model 2 | 0.001 (−0.031;0.034) | − | −0.013 (−0.051;0.026) | −0.009 (−0.044;0.026) | −0.010 (−0.046;0.026) |
| **ICA IMT_max_^m20^** |  |  |  |  |  |  |  |
|  | p75 | Model 1 | −0.028 (−0.058;0.003) | − | −0.028 (−0.064;0.008) | −0.038 (−0.070;−0.006) | −0.043 (−0.076;−0.011) |
|  |  | Model 2 | −0.015 (−0.048;0.019) | − | −0.007 (−0.046;0.032) | −0.021 (−0.057;0.014) | −0.033 (−0.070;0.004) |

Model 1: Adjustments for sex and age; Model 2: Model 1 plus physical activity, education, smoking, latitude (categorical) and diet (continuous);

m, missing values

^a^ for women cut-off >10**−**<20g/day;

^b^: for women cut-off>20g/day;

Number of observations for each analysis: IMT_mean,_ Model 1, n=3,252; Model 2, n=3,211; IMT_max_, CC-IMT_mean_ and CC-IMT_max_: Model 1, n=3,260; Model 2, n=3,219; Bif-IMT_mean_ and Bif-IMT_max_: Model 1, n=3,249; Model 2, n=3,208; ICA-IMT_mean_ and ICA-IMT_max_: Model 1, n=3,242; Model 2, n=3,201

Fig 1 (A, B): Dose-response relationships between alcohol consumption and each of the considered measurements of C-IMT (p75) at baseline (A) and progression (B). Solid lines: Restricted cubic splines adjusted for sex, age, physical activity, smoking, diet, and latitude, with knots located at fixed points of g/d of alcohol consumption (4, 10, 20, 30). Dashed lines: 95% CI. 4 g/day was used as a reference point. P for nonlinearity was obtained testing the nullity of the coefficients associated with the second, third and fourth spline basis. For a better readability of the graphs, we excluded participants with alcohol consumption >50g/d.

Table 4: Median and 75^th^ percentile differences (95% CI) of baseline C-IMT in relation to different alcohol consumption categories. Results for men (n=1,761) and women (n=1,923) included in the IMPROVE study.

| **IMT BASELINE** | | | | **Abstainers**  **(0g/d)** | **Very low**  **(0−5 g/d)** | **Low**  **(>5−10g/d)** | **Moderate**  **(>10−30g/d) ^a^** | **High**  **(>30g/d) ^b^** |
| --- | --- | --- | --- | --- | --- | --- | --- | --- |
|  |  |  |  | **β_1_ (95%CI)** | **REF** | **β_1_ (95%CI)** | **β_1_ (95%CI)** | **β_1_ (95%CI)** |
| **IMT_mean_** | Men **^m2^** |  |  |  |  |  |  |  |
|  |  | p50 | Model 1 | −0.07 (−0.12;−0.02) | - | −0.04 (−0.1;0.03) | −0.05 (−0.11;0) | −0.09 (−0.14;−0.04) |
|  |  |  | Model 2 | −0.03 (−0.07;0.02) | - | −0.01 (−0.07;0.04) | −0.03 (−0.07;0.02) | −0.04 (−0.08;0.01) |
|  |  |  |  |  |  |  |  |  |
|  |  | p75 | Model 1 | −0.06 (−0.13;0.01) | - | 0.00 (−0.08;0.08) | −0.05 (−0.12;0.02) | −0.05 (−0.12;0.02) |
|  |  |  | Model 2 | −0.02 (−0.09;0.05) | - | 0.02 (−0.06;0.10) | −0.01 (−0.08;0.06) | −0.01 (−0.08;0.07) |
|  | Women |  |  |  |  |  |  |  |
|  |  | p50 | Model 1 | −0.06 (−0.1;−0.02) | - | −0.05 (−0.10;−0.01) | −0.08 (−0.12;−0.04) | −0.08 (−0.12;−0.03) |
|  |  |  | Model 2 | −0.01 (−0.05;0.02) | - | 0.00 (−0.05;0.04) | −0.03 (−0.07;0.02) | −0.01 (−0.05;0.04) |
|  |  |  |  |  |  |  |  |  |
|  |  | p75 | Model 1 | −0.06 (−0.12;−0.01) | - | −0.07 (−0.13;−0.01) | −0.08 (−0.14;−0.02) | −0.07 (−0.14;−0.01) |
|  |  |  | Model 2 | −0.01 (−0.07;0.05) | - | 0.00 (−0.07;0.07) | −0.01 (−0.08;0.06) | −0.02 (−0.09;0.06) |
| **IMT_max_** | Men **^m2^** |  |  |  |  |  |  |  |
|  |  | p50 | Model 1 | −0.47 (−0.7;−0.24) | - | −0.27 (−0.54;0.00) | −0.38 (−0.61;−0.14) | −0.46 (−0.69;−0.23) |
|  |  |  | Model 2 | −0.35 (−0.56;−0.13) | - | −0.27 (−0.52;−0.02) | −0.25 (−0.47;−0.03) | −0.32 (−0.54;−0.10) |
|  |  |  |  |  |  |  |  |  |
|  |  | p75 | Model 1 | −0.35 (−0.62;−0.07) | - | −0.05 (−0.37;0.27) | −0.4 (−0.68;−0.13) | −0.32 (−0.59;−0.04) |
|  |  |  | Model 2 | −0.13 (−0.39;0.12) | - | 0.09 (−0.21;0.38) | −0.12 (−0.38;0.14) | −0.02 (−0.28;0.25) |
|  | Women |  |  |  |  |  |  |  |
|  |  | p50 | Model 1 | −0.29 (−0.49;−0.09) | - | −0.27 (−0.50;−0.03) | −0.40 (−0.62;−0.18) | −0.37 (−0.61;−0.13) |
|  |  |  | Model 2 | −0.11 (−0.30;0.08) | - | −0.02 (−0.25;0.21) | −0.15 (−0.37;0.07) | −0.10 (−0.34;0.13) |
|  |  |  |  |  |  |  |  |  |
|  |  | p75 | Model 1 | −0.38 (−0.62;−0.14) | - | −0.29 (−0.57;0.00) | −0.44 (−0.72;−0.17) | −0.25 (−0.54;0.04) |
|  |  |  | Model 2 | −0.23 (−0.50;0.04) | - | −0.14 (−0.45;0.18) | −0.25 (−0.56;0.05) | 0.00 (−0.33;0.32) |
| **CC−IMT_mean_** | Men **^m4^** |  |  |  |  |  |  |  |
|  |  | p50 | Model 1 | −0.03 (−0.06;0.00) | - | −0.01 (−0.05;0.02) | −0.03 (−0.06;0.00) | −0.04 (−0.07;−0.01) |
|  |  |  | Model 2 | 0.00 (−0.03;0.02) | - | 0.01 (−0.02;0.05) | −0.01 (−0.04;0.02) | −0.01 (−0.04;0.02) |
|  |  |  |  |  |  |  |  |  |
|  |  | p75 | Model 1 | −0.07 (−0.12;−0.01) | - | −0.02 (−0.08;0.04) | −0.06 (−0.11;0.00) | −0.06 (−0.11;−0.01) |
|  |  |  | Model 2 | −0.05 (−0.10;−0.01) | - | −0.02 (−0.07;0.03) | −0.06 (−0.11;−0.02) | −0.05 (−0.10;0.00) |
|  | Women |  |  |  |  |  |  |  |
|  |  | p50 | Model 1 | −0.02 (−0.05;0.00) | - | −0.02 (−0.04;0.01) | −0.03 (−0.06;−0.01) | −0.02 (−0.04;0.01) |
|  |  |  | Model 2 | 0.00 (−0.02;0.02) | - | −0.01 (−0.03;0.02) | −0.01 (−0.04;0.02) | 0.01 (−0.02;0.04) |
|  |  |  |  |  |  |  |  |  |
|  |  | p75 | Model 1 | −0.03 (−0.06;0.00) | - | −0.03 (−0.07;0.01) | −0.04 (−0.08;−0.01) | −0.03 (−0.07;0.01) |
|  |  |  | Model 2 | 0.00 (−0.04;0.03) | - | 0.00 (−0.04;0.04) | −0.02 (−0.05;0.02) | 0.01 (−0.03;0.05) |
| **Bif−IMT_mean_** | Men **^m8^** |  |  |  |  |  |  |  |
|  |  | p50 | Model 1 | −0.13 (−0.22;−0.03) | - | −0.03 (−0.14;0.08) | −0.15 (−0.25;−0.05) | −0.15 (−0.24;−0.05) |
|  |  |  | Model 2 | −0.02 (−0.12;0.07) | - | 0.03 (−0.08;0.13) | −0.05 (−0.14;0.05) | −0.05 (−0.15;0.04) |
|  |  |  |  |  |  |  |  |  |
|  |  | p75 | Model 1 | −0.10 (−0.24;0.03) | - | −0.04 (−0.2;0.11) | −0.13 (−0.27;0.00) | −0.14 (−0.27;−0.01) |
|  |  |  | Model 2 | −0.02 (−0.17;0.12) | - | 0.05 (−0.12;0.21) | −0.01 (−0.16;0.13) | −0.05 (−0.20;0.10) |
|  | Women **^m14^** |  |  |  |  |  |  |  |
|  |  | p50 | Model 1 | −0.12 (−0.20;−0.05) | - | −0.10 (−0.19;−0.01) | −0.17 (−0.26;−0.08) | −0.14 (−0.23;−0.05) |
|  |  |  | Model 2 | −0.05 (−0.12;0.03) | - | −0.03 (−0.12;0.06) | −0.07 (−0.16;0.02) | −0.03 (−0.13;0.06) |
|  |  |  |  |  |  |  |  |  |
|  |  | p75 | Model 1 | −0.11 (−0.24;0.01) | - | −0.14 (−0.29;0.00) | −0.19 (−0.33;−0.05) | −0.22 (−0.37;−0.07) |
|  |  |  | Model 2 | −0.03 (−0.16;0.09) | - | −0.07 (−0.22;0.09) | −0.09 (−0.24;0.05) | −0.11 (−0.26;0.05) |
| **ICA IMT_mean_ IMT_mean_** | Men **^m16^** |  |  |  |  |  |  |  |
|  |  | p50 | Model 1 | −0.12 (−0.19;−0.04) | - | −0.11 (−0.19;−0.02) | −0.12 (−0.19;−0.04) | −0.16 (−0.24;−0.09) |
|  |  |  | Model 2 | −0.08 (−0.16;0.00) | - | −0.06 (−0.15;0.03) | −0.07 (−0.16;0.01) | −0.10 (−0.18;−0.01) |
|  |  |  |  |  |  |  |  |  |
|  |  | p75 | Model 1 | −0.10 (−0.25;0.04) | - | −0.07 (−0.24;0.09) | −0.07 (−0.21;0.08) | −0.10 (−0.24;0.04) |
|  |  |  | Model 2 | −0.05 (−0.19;0.10) | - | 0.01 (−0.16;0.17) | −0.03 (−0.18;0.11) | 0.00 (−0.15;0.15) |
|  | Women **^m18^** |  |  |  |  |  |  |  |
|  |  | p50 | Model 1 | −0.04 (−0.08;0.01) | - | −0.05 (−0.10;0.01) | −0.06 (−0.12;−0.01) | −0.06 (−0.12;0.00) |
|  |  |  | Model 2 | 0.02 (−0.03;0.07) | - | 0.00 (−0.06;0.06) | 0.01 (−0.05;0.07) | 0.01 (−0.05;0.07) |
|  |  |  |  |  |  |  |  |  |
|  |  | p75 | Model 1 | −0.09 (−0.19;0.02) | - | −0.07 (−0.2;0.05) | −0.1 (−0.21;0.02) | −0.11 (−0.24;0.01) |
|  |  |  | Model 2 | −0.03 (−0.15;0.08) | - | 0.01 (−0.13;0.15) | −0.04 (−0.17;0.09) | −0.05 (−0.19;0.10) |
| **CC−IMT_max_** | Men **^m4^** |  |  |  |  |  |  |  |
|  |  | p50 | Model 1 | −0.05 (−0.12;0.02) | - | 0.01 (−0.08;0.09) | −0.03 (−0.1;0.04) | −0.07 (−0.14;0.00) |
|  |  |  | Model 2 | −0.04 (−0.11;0.04) |  | 0.01 (−0.07;0.10) | −0.04 (−0.12;0.03) | −0.04 (−0.12;0.03) |
|  |  |  |  |  | - |  |  |  |
|  |  | p75 | Model 1 | −0.11 (−0.26;0.03) | - | 0.05 (−0.11;0.21) | −0.07 (−0.21;0.07) | −0.05 (−0.19;0.1) |
|  |  |  | Model 2 | −0.03 (−0.17;0.11) |  | 0.10 (−0.07;0.26) | 0.00 (−0.15;0.14) | 0.03 (−0.12;0.17) |
|  | Women |  |  |  | - |  |  |  |
|  |  | p50 | Model 1 | −0.02 (−0.06;0.02) | - | −0.02 (−0.07;0.03) | −0.04 (−0.09;0.01) | −0.02 (−0.07;0.03) |
|  |  |  | Model 2 | 0.01 (−0.03;0.05) |  | −0.01 (−0.05;0.04) | −0.01 (−0.06;0.03) | 0.01 (−0.03;0.06) |
|  |  |  |  |  | - |  |  |  |
|  |  | p75 | Model 1 | −0.03 (−0.13;0.06) | - | −0.01 (−0.12;0.10) | −0.08 (−0.19;0.02) | −0.04 (−0.15;0.07) |
|  |  |  | Model 2 | 0.02 (−0.09;0.12) |  | 0.04 (−0.08;0.16) | −0.03 (−0.15;0.08) | 0.01 (−0.11;0.13) |
| **Bif−IMT_max_** | Men **^m7^** |  |  |  | - |  |  |  |
|  |  | p50 | Model 1 | −0.28 (−0.47;−0.09) | - | −0.11 (−0.34;0.11) | −0.27 (−0.46;−0.08) | −0.35 (−0.54;−0.15) |
|  |  |  | Model 2 | −0.06 (−0.25;0.12) |  | −0.01 (−0.23;0.20) | −0.02 (−0.21;0.17) | −0.08 (−0.27;0.11) |
|  |  |  |  |  | - |  |  |  |
|  |  | p75 | Model 1 | −0.40 (−0.68;−0.11) | - | −0.10 (−0.43;0.23) | −0.34 (−0.62;−0.05) | −0.38 (−0.67;−0.09) |
|  |  |  | Model 2 | −0.13 (−0.42;0.16) |  | 0.09 (−0.24;0.42) | −0.06 (−0.35;0.23) | −0.07 (−0.37;0.23) |
|  | Women **^m14^** |  |  |  | - |  |  |  |
|  |  | p50 | Model 1 | −0.20 (−0.38;−0.02) | - | −0.18 (−0.39;0.02) | −0.33 (−0.53;−0.13) | −0.24 (−0.45;−0.03) |
|  |  |  | Model 2 | −0.04 (−0.21;0.12) |  | −0.01 (−0.21;0.18) | −0.11 (−0.3;0.08) | −0.07 (−0.27;0.13) |
|  |  |  |  |  | - |  |  |  |
|  |  | p75 | Model 1 | −0.25 (−0.49;−0.01) | - | −0.26 (−0.54;0.03) | −0.33 (−0.6;−0.06) | −0.29 (−0.57;−0.01) |
|  |  |  | Model 2 | −0.08 (−0.31;0.15) |  | −0.07 (−0.34;0.2) | −0.15 (−0.41;0.11) | −0.12 (−0.39;0.16) |
| **ICA IMT_max_** | Men **^m16^** |  |  |  | - |  |  |  |
|  |  | p50 | Model 1 | −0.17 (−0.36;0.01) | - | −0.13 (−0.35;0.09) | −0.20 (−0.38;−0.01) | −0.25 (−0.44;−0.06) |
|  |  |  | Model 2 | −0.07 (−0.25;0.12) |  | −0.04 (−0.26;0.17) | −0.06 (−0.25;0.13) | −0.08 (−0.27;0.12) |
|  |  |  |  |  | - |  |  |  |
|  |  | p75 | Model 1 | −0.21 (−0.51;0.09) | - | −0.12 (−0.48;0.23) | −0.14 (−0.44;0.17) | −0.19 (−0.49;0.12) |
|  |  |  | Model 2 | −0.09 (−0.38;0.20) |  | −0.05 (−0.38;0.28) | −0.06 (−0.35;0.23) | 0.01 (−0.29;0.31) |
|  | Women **^m18^** |  |  |  | - |  |  |  |
|  |  | p50 | Model 1 | −0.09 (−0.21;0.03) | - | −0.09 (−0.23;0.05) | −0.15 (−0.29;−0.02) | −0.09 (−0.23;0.06) |
|  |  |  | Model 2 | 0.06 (−0.06;0.17) |  | 0.06 (−0.07;0.19) | 0.03 (−0.10;0.16) | 0.08 (−0.06;0.22) |
|  |  |  |  |  |  |  |  |  |
|  |  | p75 | Model 1 | −0.11 (−0.37;0.14) | - | −0.01 (−0.31;0.29) | −0.17 (−0.45;0.12) | −0.13 (−0.44;0.17) |
|  |  |  | Model 2 | 0.02 (−0.27;0.31) | - | 0.10 (−0.24;0.44) | −0.04 (−0.37;0.28) | 0.03 (−0.32;0.38) |

Model 1: Adjustments for sex and age; Model 2: adjusted for model 1 plus physical activity, education, smoking, latitude (categorical) and diet (continuous);

m, missing values

^a^ for women cut-off >10**−**<20g/day;

^b^ for women cut-off>20g/day.

Number of observations for each analysis: In women: IMT_mean,_ IMT_max_, CC-IMT_mean_ and CC-IMT_max_ Model 1, n=1,923; Model 2, n=1,898; Bif-IMT_mean_ and Bif-IMT_max_: Model 1, n=1,923; Model 2, n=1,884; ICA-IMT_mean_ and ICA-IMT_max_: Model 1, n=1,905; Model 2, n=1,880. In men: IMT_mean_ and IMT_max_: Model 1, n=1,759; Model 2, n=1,737; CC-IMT_mean_ and CC-IMT_max_ Model 1, n=1,757; Model 2, n=1,735; Bif-IMT_mean_ and Bif-IMT_max_: Model 1, n=1,754; Model 2, n=1,732; ICA-IMT_mean_ and ICA-IMT_max_: Model 1, n=1,745; Model 2, n=1,724.

Table 5: Median and 75^th^ percentile differences (95% CI) of C-IMT progression in relation to different alcohol consumption categories. Results are stratified by sex and include (1,545male and and 1,707 female participants of the IMPROVE study for whom follow-up data on C-IMT were available.

|  |  |  |  | **Abstainers**  **(0g/d)** | **Very low**  **(0−5 g/d)** | **Low**  **(>5−10g/d)** | **Moderate**  **(>10−30g/d) ^a^** | **High**  **(>30g/d) ^b^** |
| --- | --- | --- | --- | --- | --- | --- | --- | --- |
| **IMT PROGRESSION** | |  |  |  |  |  |  |  |
|  |  |  |  | **β_1_ (95%CI)** | **REF** | **β_1_ (95%CI)** | **β_1_ (95%CI)** | **β_1_ (95%CI)** |
| **IMT_mean_** | Men **^m7^** |  |  |  |  |  |  |  |
|  |  | p50 | Model 1 | −0.001 (−0.009;0.007) | - | −0.002 (−0.012;0.007) | −0.008 (−0.017;0.000) | −0.010 (−0.018;−0.002) |
|  |  |  | Model 2 | 0.000 (−0.008;0.009) | - | −0.001 (−0.01;0.009) | −0.004 (−0.012;0.004) | −0.007 (−0.015;0.002) |
|  |  |  |  |  |  |  |  |  |
|  |  | p75 | Model 1 | −0.002 (−0.011;0.007) | - | 0.006 (−0.004;0.017) | −0.010 (−0.019;−0.001) | −0.008 (−0.018;0.001) |
|  |  |  | Model 2 | −0.001 (−0.01;0.007) | - | 0.004 (−0.006;0.014) | −0.009 (−0.018;0.00) | −0.005 (−0.014;0.004) |
|  | Women **^m3^** |  |  |  |  |  |  |  |
|  |  | p50 | Model 1 | −0.009 (−0.016;−0.002) | - | −0.016 (−0.024;−0.008) | −0.009 (−0.017;−0.002) | −0.008 (−0.016;0.001) |
|  |  |  | Model 2 | −0.010 (−0.017;−0.003) | - | −0.014 (−0.022;−0.006) | −0.009 (−0.016;−0.001) | −0.009 (−0.018;−0.001) |
|  |  |  |  |  |  |  |  |  |
|  |  | p75 | Model 1 | −0.006 (−0.014;0.002) | - | −0.014 (−0.024;−0.004) | −0.007 (−0.016;0.003) | −0.007 (−0.017;0.002) |
|  |  |  | Model 2 | −0.006 (−0.015;0.003) | - | −0.011 (−0.022;−0.001) | −0.005 (−0.015;0.005) | −0.006 (−0.017;0.004) |
| **IMT_max_** | Men **^m2^** |  |  |  |  |  |  |  |
|  |  | p50 | Model 1 | 0.026 (−0.005;0.057) | - | 0.024 (−0.012;0.061) | 0.023 (−0.009;0.054) | 0.022 (−0.009;0.054) |
|  |  |  | Model 2 | 0.010 (−0.020;0.041) | - | 0.012 (−0.023;0.046) | 0.012 (−0.019;0.042) | 0.008 (−0.023;0.040) |
|  |  |  |  |  |  |  |  |  |
|  |  | p75 | Model 1 | 0.005 (−0.041;0.05) | - | −0.007 (−0.06;0.047) | −0.009 (−0.055;0.037) | −0.012 (−0.057;0.034) |
|  |  |  | Model 2 | 0.029 (−0.02;0.078) | - | 0.009 (−0.048;0.065) | 0.013 (−0.036;0.063) | 0.017 (−0.034;0.067) |
|  | Women |  |  |  |  |  |  |  |
|  |  | p50 | Model 1 | −0.003 (−0.027;0.020) | - | −0.033 (−0.061;−0.005) | −0.008 (−0.035;0.018) | −0.013 (−0.041;0.016) |
|  |  |  | Model 2 | −0.002 (−0.027;0.022) | - | −0.015 (−0.044;0.014) | 0.001 (−0.027;0.029) | −0.006 (−0.036;0.024) |
|  |  |  |  |  |  |  |  |  |
|  |  | p75 | Model 1 | 0.000 (−0.041;0.041) | - | −0.035 (−0.083;0.013) | −0.025 (−0.071;0.021) | −0.030 (−0.078;0.019) |
|  |  |  | Model 2 | 0.001 (−0.040;0.041) | - | −0.026 (−0.074;0.022) | −0.010 (−0.056;0.036) | −0.015 (−0.064;0.034) |
| **CC−IMT_mean_** | Men **^m2^** |  |  |  |  |  |  |  |
|  |  | p50 | Model 1 | −0.001 (−0.007;0.005) | - | −0.003 (−0.010;0.004) | −0.006 (−0.012;0.000) | −0.006 (−0.012;0.000) |
|  |  |  | Model 2 | 0.001 (−0.005;0.006) | - | −0.003 (−0.009;0.003) | −0.003 (−0.008;0.003) | −0.003 (−0.009;0.002) |
|  |  |  |  |  |  |  |  |  |
|  |  | p75 | Model 1 | 0.002 (−0.005;0.009) | - | 0.001 (−0.007;0.009) | −0.005 (−0.012;0.002) | −0.006 (−0.013;0.000) |
|  |  |  | Model 2 | 0.002 (−0.005;0.010) | - | 0.001 (−0.007;0.010) | −0.002 (−0.009;0.005) | −0.003 (−0.011;0.004) |
|  | Women |  |  |  |  |  |  |  |
|  |  |  | Model 1 | 0.003 (−0.002;0.008) | - | −0.001 (−0.007;0.005) | 0.000 (−0.005;0.006) | 0.004 (−0.002;0.010) |
|  |  |  | Model 2 | 0.002 (−0.003;0.006) | - | 0.001 (−0.004;0.006) | 0.001 (−0.004;0.006) | 0.004 (−0.001;0.010) |
|  |  |  |  |  |  |  |  |  |
|  |  | p75 | Model 1 | −0.003 (−0.009;0.004) | - | −0.007 (−0.015;0.001) | −0.006 (−0.014;0.001) | −0.003 (−0.011;0.005) |
|  |  |  | Model 2 | −0.002 (−0.009;0.005) | - | −0.004 (−0.011;0.004) | −0.003 (−0.011;0.004) | −0.002 (−0.010;0.006) |
| **Bif−IMT_mean_** | Men **^m4^** |  |  |  |  |  |  |  |
|  |  | p50 | Model 1 | −0.005 (−0.02;0.009) | - | −0.013 (−0.029;0.004) | −0.019 (−0.034;−0.005) | −0.020 (−0.034;−0.005) |
|  |  |  | Model 2 | −0.002 (−0.016;0.013) | - | −0.004 (−0.021;0.013) | −0.018 (−0.033;−0.003) | −0.014 (−0.029;0.001) |
|  |  |  |  |  |  |  |  |  |
|  |  | p75 | Model 1 | −0.003 (−0.024;0.017) | - | 0.012 (−0.011;0.036) | −0.019 (−0.04;0.001) | −0.014 (−0.034;0.006) |
|  |  |  | Model 2 | 0.000 (−0.022;0.021) | - | 0.008 (−0.017;0.032) | −0.012 (−0.034;0.01) | −0.009 (−0.031;0.014) |
|  | Women **^m9^** |  |  |  |  |  |  |  |
|  |  | p50 | Model 1 | −0.020 (−0.034;−0.006) | - | −0.023 (−0.04;−0.007) | −0.021 (−0.037;−0.005) | −0.019 (−0.035;−0.002) |
|  |  |  | Model 2 | −0.018 (−0.032;−0.004) | - | −0.020 (−0.036;−0.003) | −0.019 (−0.035;−0.003) | −0.023 (−0.039;−0.006) |
|  |  |  |  |  |  |  |  |  |
|  |  | p75 | Model 1 | −0.012 (−0.032;0.008) | - | −0.020 (−0.043;0.004) | −0.015 (−0.038;0.007) | −0.014 (−0.038;0.009) |
|  |  |  | Model 2 | −0.011 (−0.029;0.008) | - | −0.016 (−0.038;0.006) | −0.012 (−0.033;0.01) | −0.016 (−0.039;0.006) |
| **ICA IMT_mean_** | Men **^m16^** |  |  |  |  |  |  |  |
|  |  | p50 | Model 1 | −0.005 (−0.015;0.005) | - | 0.004 (−0.008;0.016) | −0.007 (−0.018;0.003) | −0.009 (−0.02;0.001) |
|  |  |  | Model 2 | −0.001 (−0.012;0.010) | - | 0.007 (−0.005;0.019) | −0.003 (−0.014;0.008) | −0.003 (−0.014;0.008) |
|  |  | p75 | Model 1 | −0.014 (−0.035;0.008) | - | 0.012 (−0.013;0.037) | −0.017 (−0.038;0.005) | −0.012 (−0.033;0.01) |
|  |  |  | Model 2 | −0.010 (−0.032;0.013) | - | 0.018 (−0.008;0.044) | −0.009 (−0.032;0.013) | −0.008 (−0.031;0.016) |
|  | Women **^m4^** |  |  |  |  |  |  |  |
|  |  | p50 | Model 1 | −0.013 (−0.02;−0.005) | - | −0.011 (−0.02;−0.003) | −0.014 (−0.022;−0.006) | −0.010 (−0.019;−0.002) |
|  |  |  | Model 2 | −0.012 (−0.02;−0.004) | - | −0.01 (−0.019;0.00) | −0.013 (−0.022;−0.004) | −0.008 (−0.018;0.001) |
|  |  |  |  |  |  |  |  |  |
|  |  | p75 | Model 1 | −0.007 (−0.019;0.005) | - | −0.012 (−0.026;0.002) | −0.010 (−0.024;0.003) | −0.005 (−0.019;0.009) |
|  |  |  | Model 2 | −0.007 (−0.022;0.008) | - | −0.011 (−0.028;0.007) | −0.009 (−0.025;0.008) | −0.004 (−0.022;0.014) |
| **CC−IMT_max_** | Men **^m2^** |  |  |  |  |  |  |  |
|  |  | p50 | Model 1 | 0.005 (−0.01;0.019) | - | −0.002 (−0.019;0.015) | −0.003 (−0.018;0.012) | −0.011 (−0.026;0.003) |
|  |  |  | Model 2 | 0.000 (−0.014;0.014) | - | −0.007 (−0.023;0.009) | −0.005 (−0.019;0.009) | −0.010 (−0.024;0.005) |
|  |  |  |  |  |  |  |  |  |
|  |  | p75 | Model 1 | 0.004 (−0.021;0.029) | - | −0.027 (−0.056;0.002) | −0.015 (−0.04;0.010) | −0.022 (−0.047;0.003) |
|  |  |  | Model 2 | 0.012 (−0.011;0.036) | - | −0.011 (−0.038;0.015) | 0.000 (−0.023;0.024) | −0.008 (−0.032;0.016) |
|  | Women |  |  |  |  |  |  |  |
|  |  | p50 | Model 1 | 0.002 (−0.007;0.012) | - | 0.005 (−0.006;0.016) | −0.002 (−0.013;0.009) | 0.000 (−0.011;0.012) |
|  |  |  | Model 2 | 0.003 (−0.006;0.012) | - | 0.008 (−0.002;0.019) | −0.001 (−0.011;0.010) | 0.003 (−0.008;0.014) |
|  |  |  |  |  |  |  |  |  |
|  |  | p75 | Model 1 | −0.003 (−0.019;0.012) | - | −0.001 (−0.019;0.018) | −0.012 (−0.029;0.006) | −0.008 (−0.026;0.011) |
|  |  |  | Model 2 | −0.005 (−0.022;0.012) | - | 0.002 (−0.018;0.022) | −0.013 (−0.032;0.006) | −0.008 (−0.029;0.012) |
| **Bif−IMT_max_** | Men **^m4^** |  |  |  |  |  |  |  |
|  |  | p50 | Model 1 | 0.003 (−0.025;0.031) | - | 0.000 (−0.033;0.032) | −0.001 (−0.029;0.027) | −0.001 (−0.029;0.027) |
|  |  |  | Model 2 | 0.024 (−0.004;0.052) | - | 0.01 (−0.022;0.042) | 0.019 (−0.009;0.047) | 0.018 (−0.010;0.047) |
|  |  |  |  |  |  |  |  |  |
|  |  | p75 | Model 1 | −0.001 (−0.049;0.046) | - | −0.002 (−0.058;0.054) | −0.02 (−0.068;0.028) | −0.03 (−0.078;0.018) |
|  |  |  | Model 2 | 0.018 (−0.032;0.068) | - | −0.002 (−0.059;0.055) | 0 (−0.051;0.05) | 0.001 (−0.051;0.052) |
|  | Women |  |  |  |  |  |  |  |
|  |  | p50 | Model 1 | −0.002 (−0.026;0.023) | - | −0.002 (−0.032;0.028) | −0.003 (−0.031;0.025) | 0.001 (−0.029;0.031) |
|  |  |  | Model 2 | −0.005 (−0.030;0.020) | - | 0.000 (−0.030;0.030) | −0.003 (−0.032;0.025) | 0.005 (−0.026;0.035) |
|  |  |  |  |  |  |  |  |  |
|  |  | p75 | Model 1 | −0.007 (−0.05;0.037) | - | −0.034 (−0.087;0.018) | −0.031 (−0.081;0.019) | 0.003 (−0.05;0.055) |
|  |  |  | Model 2 | 0.000 (−0.048;0.048) | - | −0.022 (−0.079;0.036) | −0.020 (−0.074;0.035) | 0.018 (−0.04;0.077) |
| **ICA IMT_max_** | Men **^m9^** |  |  |  |  |  |  |  |
|  |  | p50 | Model 1 | −0.008 (−0.033;0.018) | - | 0.003 (−0.027;0.033) | 0.001 (−0.025;0.026) | −0.012 (−0.037;0.014) |
|  |  |  | Model 2 | −0.003 (−0.028;0.022) | - | 0.003 (−0.026;0.032) | 0.004 (−0.022;0.029) | −0.003 (−0.029;0.022) |
|  |  |  |  |  |  |  |  |  |
|  |  | p75 | Model 1 | −0.028 (−0.077;0.021) | - | 0.001 (−0.056;0.059) | −0.03 (−0.079;0.02) | −0.044 (−0.094;0.005) |
|  |  |  | Model 2 | −0.005 (−0.053;0.043) | - | 0.013 (−0.042;0.069) | −0.02 (−0.068;0.029) | −0.021 (−0.07;0.029) |
|  | Women **^m4^** |  |  |  |  |  |  |  |
|  |  | p50 | Model 1 | −0.021 (−0.038;−0.005) | - | −0.033 (−0.053;−0.014) | −0.026 (−0.044;−0.007) | −0.023 (−0.043;−0.004) |
|  |  |  | Model 2 | −0.026 (−0.043;−0.01) | - | −0.033 (−0.052;−0.013) | −0.029 (−0.048;−0.01) | −0.031 (−0.051;−0.011) |
|  |  |  |  |  |  |  |  |  |
|  |  | p75 | Model 1 | −0.025 (−0.062;0.012) | - | −0.039 (−0.084;0.006) | −0.039 (−0.081;0.003) | −0.038 (−0.083;0.007) |
|  |  |  | Model 2 | −0.024 (−0.065;0.017) | - | −0.041 (−0.090;0.008) | −0.035 (−0.081;0.012) | −0.033 (−0.083;0.016) |

Model 1: adjusted for sex and age; Model 2: adjusted for model 1 plus physical activity, education, smoking, latitude (categorical) and diet (continuous);

m, missing values

^a^ for women cut-off >10**−**<20g/day;

^b^ for women cut-off>20g/day. . Number of observations for each analysis: In women: IMT_mean,_ Model 1, n=1,704; Model 2, n=1,683; IMT_max_, CC-IMT_mean_ and CC-IMT_max_: Model 1, n=1,707; Model 2, n=1,686; Bif-IMT_mean_ and Bif-IMT_max_: Model 1, n=1,698; Model 2, n=1,677; ICA-IMT_mean_ and ICA-IMT_max_: Model 1, n=1,693; Model 2, n=1,671; In men: IMT_mean,_ Model 1, n=1,548; Model 2, n=1,528; IMT_max_, CC-IMT_mean_ and CC-IMT_max_: Model 1, n=1,553; Model 2, n=1,533; Bif-IMT_mean_ and Bif-IMT_max_: Model 1, n=1,551; Model 2, n=1,531; ICA-IMT_mean_ and ICA-IMT_max_: Model 1, n=1,549; Model 2, n=1,529

Contributors of the IMPROVE Study group:

C.R. Sirtori, S. Castelnuovo, M. Amato, B. Frigerio, A. Ravani, D. Sansaro, C. Tedesco, D. Coggi, A. Bonomi, M.J. Eriksson, J. Cooper, J. Acharya, K. Huttunen, E. Rauramaa, H Pekkarinen, I.M. Penttila, J. Törrönen, A.I. van Gessel, A.M van Roon, G.C. Teune, W.D. Kuipers, M. Bruin, A. Nicolai, P. Haarsma-Jorritsma, D.J. Mulder, H.J.G. Bilo, G.H. Smeets, J.L. Beaudeux, J.F. Kahn, V. Carreau, A. Kontush, J. Karppi, T. Nurmi, K. Nyyssönen, R. Salonen, T.P. Tuomainen, J.Tuomainen, J. Kauhanen, G. Vaudo, A. Alaeddin, D. Siepi, G. Lupattelli, E. Mannarino.
